# Gene duplication and co-evolution of G1/S transcription factor specificity in fungi are essential for optimizing cell fitness

**DOI:** 10.1371/journal.pgen.1006778

**Published:** 2017-05-15

**Authors:** Adi Hendler, Edgar M. Medina, Anastasiya Kishkevich, Mehtap Abu-Qarn, Steffi Klier, Nicolas E. Buchler, Robertus A. M. de Bruin, Amir Aharoni

**Affiliations:** 1 Department of Life Sciences and the National Institute for Biotechnology in the Negev, Ben-Gurion University of the Negev, Be’er Sheva, Israel; 2 Department of Biology, Duke University, Durham, United States; 3 Center for Genomic and Computational Biology, Duke University, Durham, United States; 4 MRC Laboratory for Molecular Cell Biology, University College London, London, United Kingdom; Stanford University School of Medicine, UNITED STATES

## Abstract

Transcriptional regulatory networks play a central role in optimizing cell survival. How DNA binding domains and *cis*-regulatory DNA binding sequences have co-evolved to allow the expansion of transcriptional networks and how this contributes to cellular fitness remains unclear. Here we experimentally explore how the complex G1/S transcriptional network evolved in the budding yeast *Saccharomyces cerevisiae* by examining different chimeric transcription factor (TF) complexes. Over 200 G1/S genes are regulated by either one of the two TF complexes, SBF and MBF, which bind to specific DNA binding sequences, SCB and MCB, respectively. The difference in size and complexity of the G1/S transcriptional network across yeast species makes it well suited to investigate how TF paralogs (SBF and MBF) and DNA binding sequences (SCB and MCB) co-evolved after gene duplication to rewire and expand the network of G1/S target genes. Our data suggests that whilst SBF is the likely ancestral regulatory complex, the ancestral DNA binding element is more MCB-like. G1/S network expansion took place by both *cis*- and *trans*- co-evolutionary changes in closely related but distinct regulatory sequences. Replacement of the endogenous SBF DNA-binding domain (DBD) with that from more distantly related fungi leads to a contraction of the SBF-regulated G1/S network in budding yeast, which also correlates with increased defects in cell growth, cell size, and proliferation.

## Author summary

Cell cycle progression is essential for the division of living cells. The G1/S transition through the cell cycle is promoted by the periodic expression of a large set of genes regulated by a G1/S transcriptional gene network. Despite extensive comparative studies in different eukaryotic organisms, little is known regarding the evolution of G1/S transcriptional network expansion and how such an expansion was important for cell division and growth rate. Here, we explored the evolution of G1/S transcriptional network in the budding yeast *S*. *cerevisiae* by examining 16 different chimeric transcription factor complexes containing DNA binding domain from different fungal species. Analysis of the 16 chimeric SBF and MBF complexes in *S*. *cerevisiae* that bind specific SCB and MCB DNA sequences, respectively, suggests that SBF is more closely related to the ancestral regulatory complex. We found that some of the chimeric SBF complexes can induce the expression of a subset of genes in *S*. *cerevisiae* that are enriched with an MCB-like element.

## Introduction

**Fig 1:**
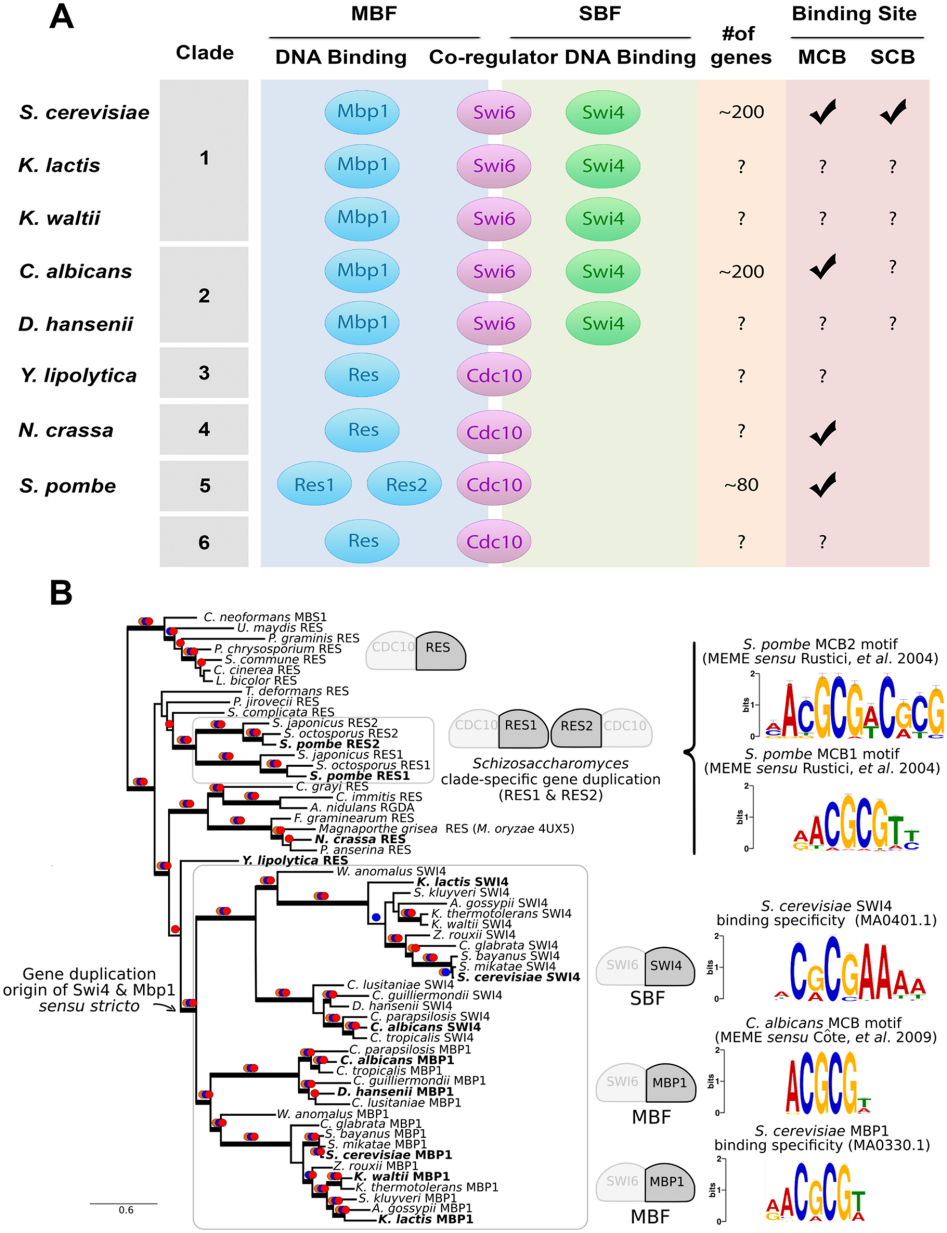
Evolution of transcription factors involved in G1/S cell cycle control in Dikarya. (A) Conservation of G1/S TF from [5] and the expansion of the gene expression network (number of genes expressed during G1/S from [1, 6]). (B) Evolutionary history of the G1/S transcription factors that bind DNA (Res, Swi4, Mbp1, outlined in black) in Dikarya. Our work is focused on the evolution of DNA-binding specificity and, thus, our analysis did not include the co-regulator (Swi6, Cdc10). Species used in our study are highlighted in bold. Consensus DNA-binding motifs for each DNA-binding protein (*S*. *cerevisiae* Swi4 and Mbp1) were obtained from JASPAR (ID shown) or reconstructed from promoter regions (1000bp upstream) of previously published target genes [4, 6] with MEME (*C*. *albicans* MCB, *S*. *pombe* MCB1 and MCB2). Maximum likelihood phylogenetic tree (RAxML, LG substitution matrix) is shown. Thick branches indicate a highly supported branch. Orange dots: RAxML RBS ≥ 70%, Blue dots: PhyML SH-aLRT ≥ 80%, Red dots: PhyML a la Bayes aLRT ≥ 90%. Details of MEME and phylogenetic analysis are in Materials & Methods.

Eukaryotic cells have evolved complex transcriptional regulatory networks to ensure faithful cell division. One example is the G1/S cell cycle network that includes a large set of co-regulated genes whose expression peaks at the G1-to-S transition. Activation of G1/S transcription promotes entry into S phase and the initiation of a new cell division cycle. Previous work has established that the regulatory mechanisms involved in controlling G1/S transcription are conserved from yeast to man [1–4]. In animals, E2F/DP is a large family of winged helix-turn-helix transcription factors that regulate G1/S target genes. In budding yeast (*S*. *cerevisiae*), the main G1/S transcription factor (TF) components, Swi4, Swi6 and Mbp1, form two heterodimer transcription factor complexes: a common Swi6 subunit plus one of the DNA binding proteins Swi4 or Mbp1 constitute SBF and MBF complexes, respectively (Fig 1A). The related components in fission yeast (*S*. *pombe*), the common Cdc10 subunits with Res1 and Res2, forms a tetramer complex [1].

In budding yeast, SBF and MBF regulate distinct branches of the G1/S transcriptional network [7–9]. The MCB (***M****luI* **C**ell-cycle **B**ox) recognition sequence *ACGCGT*, bound by Mbp1, can be found in the promoters of G1/S genes in different fungi, such as *C*. *albicans* [4] and *S*. *pombe* [6]. However, the SCB (**S**wi4 **C**ell-cycle **B**ox) recognition sequence *CRCGAAA*, bound by Swi4, is only found in budding yeast (Fig 1A). It is generally presumed that ancestral Res (the progenitor of Swi4 and Mbp1 in hemiascomycetes) bound an MCB-like motif and that SCB is the more specialized DNA-binding motif that emerged sometime after Res duplication. This scenario represents a classic case of neofunctionalization after gene duplication, where one of the paralogs (Swi4) evolves a new function and DNA-binding specificity (SCB) to regulate old and new G1/S target genes [10].

However, recent work suggests that SCB might be the ancestral motif. Yeast SBF and MBF are winged helix-turn-helix proteins that play a similar role to the E2F/DP transcription factors in animal G1/S regulation [3]. Comparative genomics shows that E2F/DP in the fungal ancestor was functionally replaced by horizontal transfer of an ancestral Res transcription factor, which has homology to a viral KilA-N domain [5]. The similar functional role and overlap in the known binding motifs of budding yeast Swi4 (*CRCGAAA*) and human E2F (*GCGSSAAA*) suggested that ancestral Res could bind the same cis-regulatory elements targeted by E2F/DP. In vivo experiments showed that Swi4 (but not Mbp1) recognized and bound heterologous E2F sites in budding yeast [5]. This result and the presence of MCB-like elements in other fungi [11] raise the possibility that ancestral Res may have bound an extended SCB/MCB-like motif (RCB, **R**es **C**ell cycle **B**ox). The ancestral Res duplicated into Swi4 and Mbp1 in the hemiascomycetes, where each paralog may have split the broader RCB into more specialized motifs (SCB and MCB) to regulate old and new G1/S genes. This scenario represents sub-functionalization after gene duplication [10].

The number of target genes in the G1/S network ranges from ~200 in budding yeast to ~80 in the distantly related fission yeast [8, 9, 12, 13]. The difference in size and complexity of the G1/S transcriptional network in yeast makes it well suited to investigate how DNA binding domains (Swi4 and Mbp1) and DNA binding sequences (SCB and MCB) co-evolved to rewire and expand the network of G1/S target genes. To address these different evolutionary scenarios and to examine *in vivo* function, we generated 16 different chimeric TFs by systematic replacements of native *S*. *cerevisiae* DBD in Mbp1 and Swi4 with orthologs from different fungal species. We show that chimeric TFs containing the DBD of distant orthologs fused to *S*. *cerevisiae* Swi4 activation domain regulate the expression of a progressively limited subset of SBF-dependent target genes in budding yeast. The subset of SBF-targets regulated by the chimeric TFs contain motifs more closely related to SCB/MCB-like motifs (RCB) consistent with a Res-like ancestor, as found in *S*. *pombe*. This suggests that network expansion took place by expanding the ancestral SBF regulon, which contained RCB motifs, via inclusion of the modern SCB motif. We found that Swi4 exhibits much higher affinity for the SCB motif suggesting an optimized binding motif for Swi4. Our case study can also be used to investigate if complex transcriptional regulatory networks are optimized by natural selection for cellular fitness or whether they are the result of evolutionary trajectories of ‘least resistance’ [14]. We show that network expansion is important for maintaining accurate cell division and normal growth rate of *S*. *cerevisiae*. More generally, our experimental analysis reveals that network expansion can depend on gradual co-evolution of DBD with diverse promoters to include genes containing new regulatory motifs for optimizing cellular fitness.

## Results

Our comparative analysis of fungal genomes [5] showed that the ancestor of Ascomycetes and Basidiomycetes had a single Res DNA-binding subunit, which presumably formed a heterodimer with Cdc10 co-regulator (*S*. *cerevisiae* Swi6); see Fig 1A. Interestingly, many Hemiascomycetes and fission yeasts have accumulated lineage-specific duplications of their G1/S transcription factors that are not shared with each other or the ancestor of most fungi (Fig 1B). The ancestral Res gene duplicated into Swi4 (SBF subunit) and Mbp1 (MBF subunit) in the ancestor of Clades 1–2 of Hemiascomycetes (e.g. *Candida*, *Kluyveromyces*, *Saccharomyces*) whereas it duplicated into Res1 and Res2 (Cdc10 tetramer) in the ancestor of the Schizosaccharomycetes (Clade 5). This raises the question of whether Swi4 or Mbp1 evolved new DNA binding specificities and gene targets upon duplication. Conversely, which DNA sequence did the ancestral Res subunit bind?

### *In vivo* testing of functional conservation of DBDs from different yeasts

**Fig 2:**
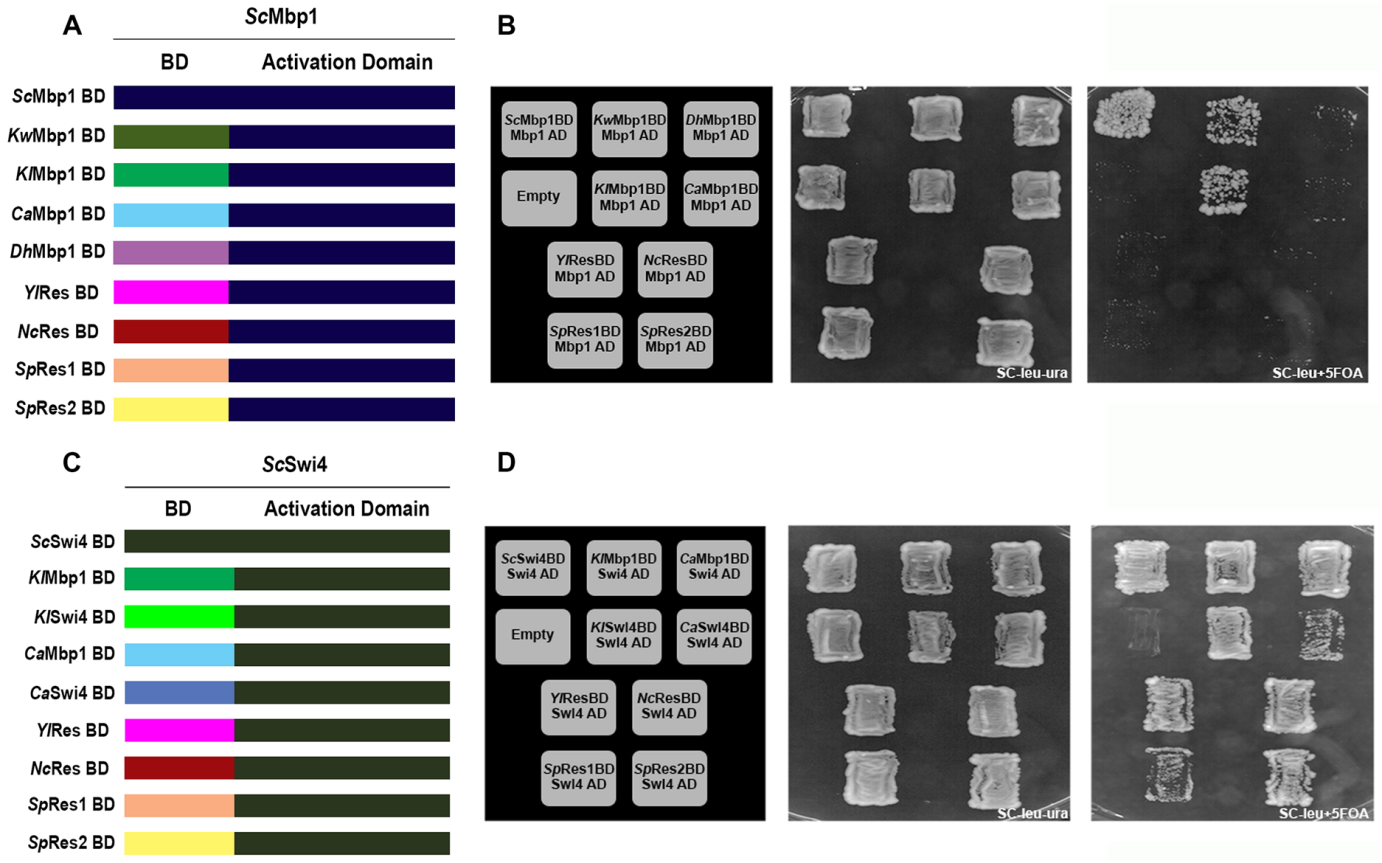
*In vivo* phenotypic analysis of chimeric Mbp1/Swi4 TF. (A) Schematic representation of the chimeric Mbp1 TFs based on the substitution of the native *Sc* Mbp1 DBD by different orthologues. Orthologues from the different clades were chosen based on the fungi phylogenetic tree (Fig 1). (B) *In vivo* phenotypic analysis of the different chimeric Mbp1s in *swi4Δmbp1Δ* strain. Substitution of ScMbp1BD by CaMbp1BD, DhMbp1BD, YlResBD, NcResBD, SpRes1BD and SpRes2BD results in non-viability of the cells, while KwMbp1BD and KlMbp1BD exhibit complementation ability. (C) Schematic representation of the chimeric Swi4 TFs based on the substitution of the native ScSwi4BD by different orthologues from the different clades based on the fungal phylogenetic tree. (D) *In vivo* phenotypic analysis of the different chimeric Swi4 TFs in *swi4Δmbp1Δ* strain. Substitution of ScSwi4BD by all orthologues across the phylogenetic tree exhibits complementation that supported yeast viability.

Our phylogenetic analysis of Mbp1 and Swi4 DBDs shows that both duplicates originated from the same duplication event from a Res ancestor (Fig 1B). We next tested DBD functional conservation through Ascomycete evolution by systematic replacements of the native *S*. *cerevisiae* Mbp1/Swi4 DBD with those from different ascomycete fungi which share high sequence similarity [11, 15, 16]. Expression of *Sc*Mbp1 or *Sc*Swi4 rescues the lethality of an *mbp1Δswi4Δ* double knockout in *S*. *cerevisiae* because a critical fraction of rate-limiting G1/S genes is expressed, e.g. *CLN2* [17]. Thus, we expect that Mbp1 and/or Swi4 chimeric TFs could rescue the lethality of an *mbp1Δswi4Δ* double knockout if these DBD can bind to critical *Sc*SCB or *Sc*MCB motifs to sufficiently activate gene expression. This complementation analysis could reveal both the specificity (MBF and/or SBF-dependent) and extent (level of rescue) of functional conservation, and provide us with a perspective on how the G1/S cis-regulatory network might have changed during the evolution of Hemiascomycetes. We generated sixteen different chimeric TFs by replacing the *Sc*Mbp1 DBD or *Sc*Swi4 DBD with homologues from representative fungal species from five Ascomycete clades, including *K*. *lactis* and *K*. *waltii* from clade 1, *D*. *hansenii* and *C*. *albicans* from clade 2, *Y*. *lipolytica* from clade 3, *N*. *crassa* from clade 4 and *S*. *pombe* from clade 5 (Fig 2A and 2C). We chose the recombination point between the DBDs and the *S*. *cerevisiae* AD at the end of the Sc DBD (in the case of Mbp1 at aa 125 and in the case of Swi4 at aa 166) based on the conservation level between the DBDs and previous structure/function analysis of recombinant *S*. *cerevisiae* Mbp1 and Swi4 DBDs [11, 15, 16]. Our rationale was to generate chimeric proteins in which the C-terminal AD domains of *Sc*Mbp1 or *Sc*Swi4 were preserved. To support our choice of recombination points, we performed IUPred analysis of Mbp1 and Swi4 [18, 19]. We found that the functional domains of the proteins (e.g. the DBD, Ankyrin repeat and the C-terminal domain [20]) are predicted to be structured while the regions connecting these domains are predicted to be unstructured (S1 Fig). This analysis supports a ball-and-string model where structural and functional regions are connected by flexible unstructured regions. The ball-and-string model is further supported by previous work that generated and tested chimeric proteins using similar recombination points between the DBD, Ankyrin repeat, and C-terminal domains [20].

These chimeric proteins were expressed from the native *Sc*Mbp1 or *Sc*Swi4 promoter on a centromeric LEU2 plasmid in *mbp1Δswi4Δ* double knock-out strain, which is rescued by the expression of *Sc*Mbp1 or *Sc*Swi4 from a centromeric URA3 plasmid (S2 Fig**)**. To generate yeast haploid strains expressing the chimeric protein as their sole source of G1/S transcription factor (TF), we selected against the URA3 rescue plasmid using 5-fluoroorotic acid (5-FOA) (S2 Fig, details are provided in Materials and Methods). The ability of a chimeric TF in a *mbp1Δswi4Δ* strain to grow on 5-FOA media indicates that the chimera binds and activates a critical subset of target G1/S genes, which includes *CLN2* [17]. We found that all strains expressing chimeric TFs with Swi4 Activation Domain (Swi4AD) fused to homologous DBD (Swi4, Mbp1, Res) from all fungi tested were viable, although to different degrees **(**Fig 2D). In contrast, only those strains expressing chimeric Mbp1AD fused to orthologous Mbp1 DBD from clade 1 were viable whereas all other DBDs (Clades 2–5) were non-viable (Fig 2B). Since the chimeric proteins are largely expressed at a similar level as the native *Sc*Mbp1 or *Sc*Swi4 protein (S3 Fig), the observed trends in viability of chimeric TFs are not explained by differences in protein abundance. Rather, they are most likely a consequence of reduced DNA binding potential.

### Most chimeric TFs with Mbp1AD cannot regulate *S*. *cerevisiae* MBF-dependent targets

The inability of the Mbp1AD chimeras outside of clade 1 DBDs to complement *Sc*Mbp1 function and rescue the *mbp1Δswi4Δ* lethality was surprising because all Swi4AD chimeras were viable. We therefore tested if the chimeric TFs with Mbp1AD can regulate the transcription of a set of G1/S targets including the MBF target *CDC21*, the SBF-MBF switch gene *TOS4*, and the SBF target *SVS1* in a *mbp1*Δ *SWI4* background [21]. We found that, as expected, the expression levels of *CDC21* and *TOS4* in strains containing clade I BD-Mbp1ADs are periodic, similar to the WT *Sc*Mbp1, but strains containing clades II-V BD-Mbp1ADs show non-periodic gene expression, similar to the *mbp1*Δ strain (S4 Fig). Surprisingly, when we analyzed the periodic expression of the Swi4 target gene, *SVS1*, we observed that the Mbp1AD chimeric TFs from clades II-V lead to deregulation of the periodic expression of *SVS1* when cells exit from G1 phase (S4 Fig). These data indicate that the Mbp1AD chimeras might interfere with the endogenous Swi4 protein ability to properly regulate SBF-dependent transcription because SBF usually releases its target promoters at this stage [22].

### Cells that rely on chimeric TFs with Swi4AD for G1/S transcriptional regulation show phenotypic defects

**Fig 3:**
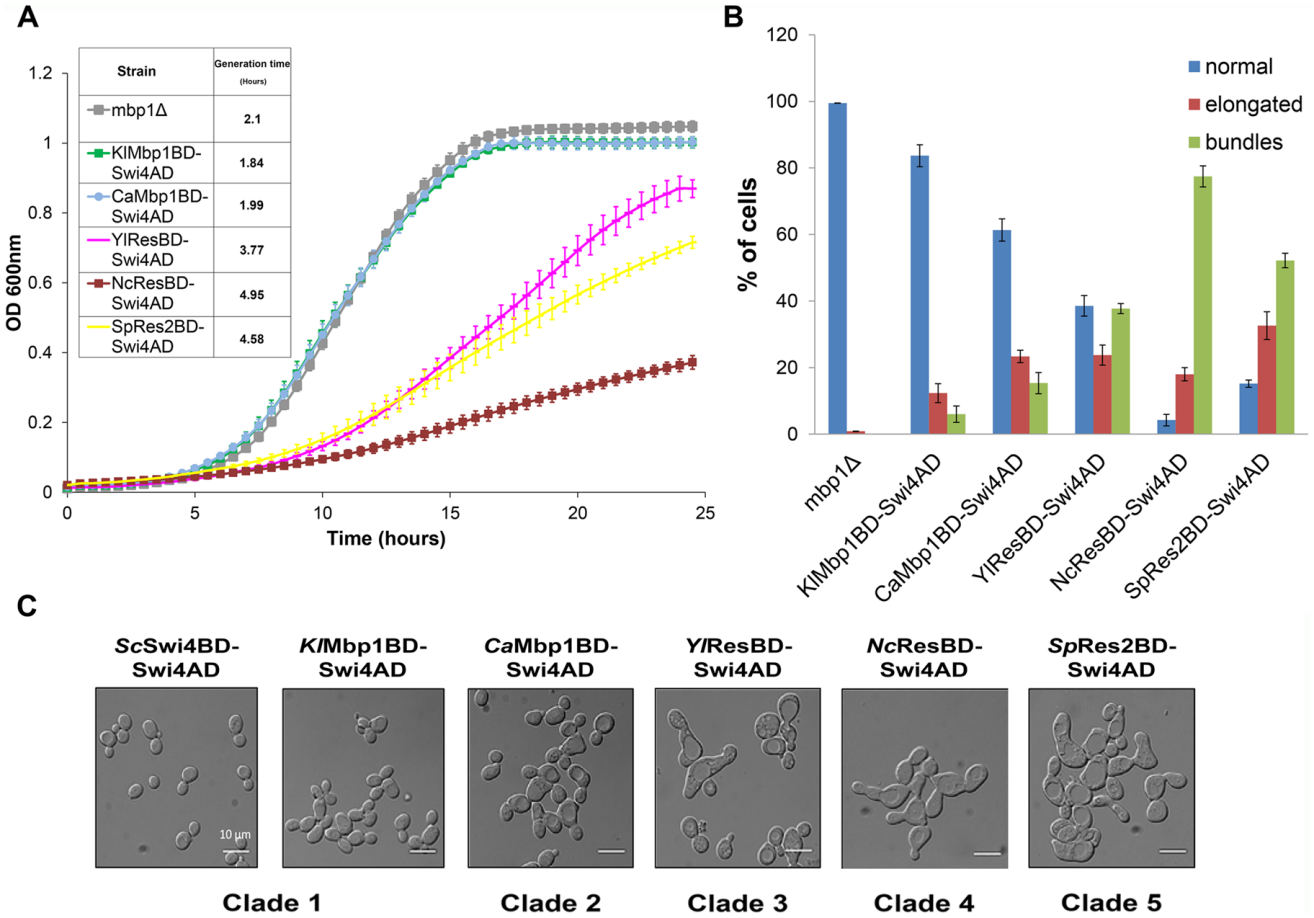
*In vivo* phenotypic analysis of chimeric Swi4 TFs. (A) Growth analysis of chimeric Swi4 TFs integrated into the genome in the background of *mbp1Δ* strain. The strains containing chimeric *Kl*Mbp1BD-Swi4 AD (green line) and *Ca*Mbp1BD-Swi4AD (light blue line) display similar growth as the *mbp1Δ* strain (grey line). Strains with chimeric *Yl*ResBD-Swi4AD (pink line), *Nc*ResBD-Swi4AD (red line) and *Sp*RES2BD-Swi4AD (yellow line) exhibit impaired growth rates relative to the *mbp1Δ* strain. (B) Quantification of the cell morphology of the different strains by light microscopy manual analysis. Each strain was analyzed in triplicates and at least 100 cells were analyzed and manually categorized (C) Strains morphology was analyzed by microscope. *Kl*Mbp1BD-Swi4 AD, *Kl*Swi4 BD-Swi4AD and *Ca*Mbp1BD-Swi4AD strains exhibit similar morphology as the *mbp1Δ* strain, while *Nc*ResBD-Swi4AD and *Sp*Res2BD-Swi4AD cells are elongated and exhibit severe defects in cell division.

We focused on chimeric TFs with Swi4AD because our data suggest that they are functional TFs that can bind a critical subset of G1/S targets. However, while all chimeric TFs with Swi4AD can rescue *mbp1Δswi4Δ* lethality, there are differences in the robustness of the rescue (Fig 2). To examine the *in vivo* complementation of the chimeric TFs with Swi4AD in more detail, we replaced the *Sc*Swi4 DBD with those from different yeasts at the endogenous locus in a *mbp1*Δ background. We reasoned that any loss of SBF-dependent regulation by the chimeric TFs with Swi4AD is more likely to result in defects in growth rate and cell morphology in a *mbp1*Δ background. Examining the phenotype of *mbp1*Δ strains expressing chimeric TFs with Swi4AD revealed that the growth rate of strains containing DBD orthologue from clades 1–2 (*Kl*Mbp1BD-Swi4AD, *Kl*Swi4BD-Swi4AD or *Ca*Mbp1BD-Swi4AD) are similar to that of the WT (Fig 3 and S5 Fig for additional strain analysis). In contrast, the strains expressing the chimeric TFs with Swi4AD containing DBD orthologue from clade 3–5 (*Yl*ResBD-Swi4AD, *Nc*ResBD-Swi4AD or *Sp*Res2BD-Swi4AD) exhibit impaired growth rate with up to a two-fold increase in doubling time (Fig 3A). In agreement with these results, we also observed impaired growth of these strains at 37°C (S5 Fig). Finally, examination of cell morphology, using light microscopy reveals that the different chimeric Swi4 TFs variants show a range of defects such as elongated cells and the formation of cell bundles due to possible defects in G2/M phase (Fig 3B–3C). Importantly, the severity of the defect in growth, temperature sensitivity and cell morphology correlated with their evolutionary distance from *Sc*. Specifically, we found that strains containing the *Yl*ResBD-Swi4AD, *Nc*ResBD-Swi4AD or *Sp*Res2BD-Swi4AD cells are elongated or created bundles of cells suggesting severe defects in cell division (S1 and S2 Movies).

### Establishing the SBF-regulon by genome wide expression analysis

Our analysis of phenotypes of cells expressing chimeric TFs with Swi4AD suggests a range of scenarios. For example, DBDs from more distantly related yeast could bind the same SBF-dependent genes as *Sc*Swi4, but activate them less robustly (e.g. decreased activation potential). Alternatively, these DBDs could regulate a different and/or smaller set of genes (e.g. changed binding specificity). To establish the extent that chimeric Swi4 TFs can restore SBF-dependent transcriptional control, we performed qPCR on candidate genes in cell cycle synchronized cells of selected strains. We focused on specific chimeras containing Mbp1/Res DBD that spans broad evolutionary distance and displays a range of cell phenotypes. We analyzed four strains containing chimeric TFs with Swi4AD from *K*. *lactis* from clade 1, *C*. *albicans* from clade 2, *N*. *crassa* from clade 4 and *S*. *pombe* from clade 5, in addition to WT and *swi4Δ* strains as controls. All chimeras were examined in the background of *MBP1* to maintain normal growth rate, cell morphology, and efficient synchronization in response to alpha-factor. Strains were synchronized with alpha-factor and gene expression was measured at five time points (0, 15, 30, 45, 60 minutes) after release. Time-points correspond to low G1/S expression levels in arrested cells at time 0, peak levels at 30 minutes and transcriptional inactivation at 60 minutes, as shown for *RNR1* (an MBF-regulated gene) and *CLN2* (an SBF-regulated gene) for chimeric strains and wild-type (S6 Fig). Based on budding index, cell cycle progression is comparable in all alpha-factor arrested and released strains. SBF-dependent gene expression of *CLN2* was strongly reduced in *swi4Δ*, although it still exhibited a weak pulse of activation after release presumably due to endogenous Mbp1. Our results demonstrate that chimeric TFs created from DBDs of more distantly related species exhibit decreasing *CLN2* expression, which is consistent with our observations regarding cell phenotypes (Fig 3 and S5 Fig). This suggests that defects in the expression of SBF-dependent genes by chimeric TFs with Swi4AD underlie the decreased cell fitness.

Based on these results, we used genome-wide expression analysis (RNA-seq) to first define the SBF-dependent regulon as those sets of genes that show significant changes in *swi4Δ* relative to wild type over time. All RNA-seq experiments were performed using three biological replicates. Strains were synchronized with alpha-factor and gene expression was measured at two time points (0, 30 minutes) after release. Potential SBF-regulated genes were identified by using likelihood ratio test (LRT) on WT versus *swi4Δ* gene expression over time, in which we compare the full model (~strain + time + strain:time) with a reduced model in which we removed strain specific differences over time (i.e., the interaction term strain:time). Genes that show differences in their pattern of expression at a 10% false discovery rate (i.e. Benjamini-Hochberg adjusted p_BH_-value of 0.1) were considered likely SBF targets (S7 Fig). Genes that change their pattern of expression in the same way in WT and *swi4Δ* were not considered significant. We ranked significance of SBF target genes by their strength of regulation based on log2 fold change in expression relative to WT.

**Fig 4:**
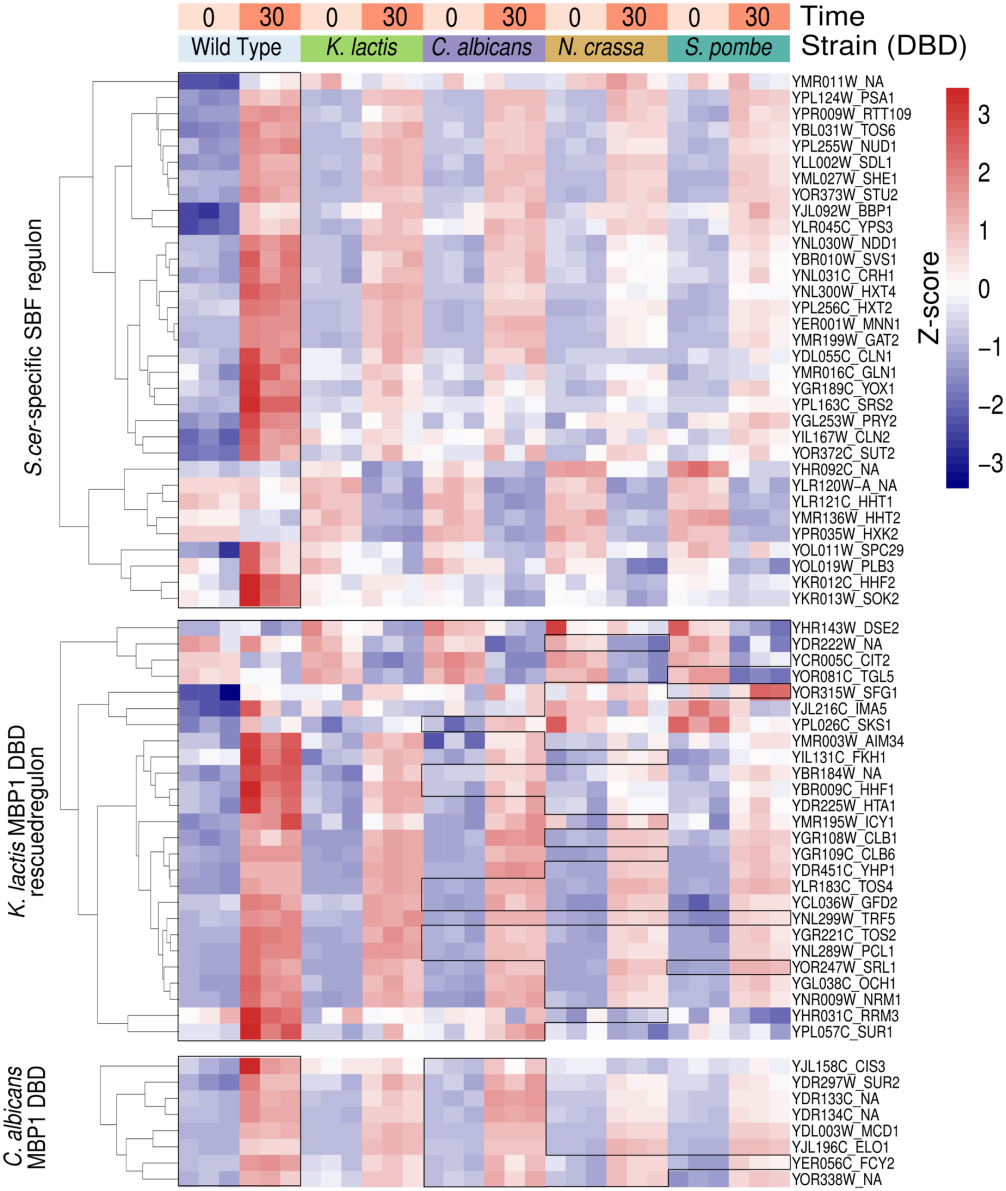
Rescue of SBF-activated gene expression by chimeric SBF TFs. Gene expression profile of SBF-activated regulon at two time points (0 and 30 mins) after release from alpha-factor block. We plot the regularized-logarithm transformed data of each gene (row) for each strain (wild-type or DBD chimeras, columns) for visualization. All RNA-seq experiments were done in triplicate. The RNA-seq expression of each gene (heatmap) is plotted as a Z-score relative to the mean and standard deviation of all values on the same row. Statistical analysis with DESeq2 was performed on the raw data. Genes that had no statistical difference between RNA-seq expression at 0 and 30 mins between wild-type and chimeras were grouped together within the same contiguous box. Genes (rows) outside the box indicate that expression was significantly different from wild-type and not rescued by DBD chimeras of the corresponding species (column). Genes were organized top-down starting with the genes from SBF-activated regulon that were only rescued by *Scer*Swi4BD (*Scer*-specific SBF regulon). We then list genes from SBF-activated regulon that were also rescued by *Kl*Mbp1 (*Klac* Mbp1DBD-rescued regulon). Genes rescued by *Kl*Mbp1DBD were usually rescued by *Ca*Mbp1BD with occasional rescue by *Nc*ResBD and *Sp*Res2BD. Last, we show those genes not rescued by *Kl*Mbp1BD, but which were rescued by *Ca*Mbp1BD with occasional rescue by *Nc*ResBD. Each module was organized using hierarchical clustering of RNA-seq profiles.

A total of 68 genes showed significant down-regulation in expression in *swi4Δ* relative to wild type, which we defined as the “SBF-activated” regulon (Fig 4 and S1 Table). Thirty of these 68 SBF-activated genes (*TOS6*, *SVS1*, *CLN1*, *MNN1*, *CLB6*, *PRY2*, *YOX1*, *CIS3*, *HHF1*, *MCD1*, *CLN2*, *BBP1*, *EXG1*, *NRM1*, *SRL1*, *HHF2*, *YPS3*, *YOL019W*, *SUR2*, *HTA1*, *HHT2*, *NDD1*, *CRH1*, *SPC29*, *STU2*, *PSA1*, *SUR1*, *NUD1*, *TOS4*, *OCH1*) are shared with the SBF targets suggested by Ferrezuelo et al. [12] and the identity of our genes suggests that our assay is indeed identifying potential SBF-activated targets. The gene that shows the strongest regulation in our SBF-activated regulon was *TOS6* (>19 fold), which was ranked second in the previous study [12] and seven SBF target genes from [12] are within our top 10 SBF target genes (*TOS6*, *SVS1*, *CLN1*, *MNN1*, *CLB6*, *PRY2*, *YOX1*). There were an additional 64 genes that showed up-regulation in expression in *swi4Δ*, which we defined as the "SBF-repressed" regulon (S7 Fig).

As expected, the highest enrichment in our SBF-activated gene set corresponds to the “cell cycle” GO term (e.g. GO:0007049 p_BH_-value 1.01E-4) and other related terms (S8 Fig). For example, the cyclin *PCL1*, which is our second most strongly differentially expressed SBF-activated gene (-3.7 log2 fold change), regulates polarized growth and morphogenesis during cell cycle and has been suggested previously to be regulated by Swi4 [23]. Some genes (*TOS6*, *HTA1*, *HHF2*, *SVS1 & PRY2*) are known to respond to alpha-factor, which was how we synchronized our cells via pheromone block-release. These genes still showed significant differences in expression between *swi4Δ* and WT.

### More distantly related chimeric TF variants appear to regulate an increasing smaller subset of SBF targets

We then performed the same RNA-seq experiments with several chimeric TFs with Swi4AD (*Kl*Mbp1BD-Swi4AD, *Ca*Mbp1BD-Swi4AD, NcResBD-Swi4AD, *Sp*Res2BD-Swi4AD), which previously had restored viability in *swi4Δmbp1Δ* but exhibited different effects on growth rate and cell morphology. The ability of a chimeric TF to rescue the gene expression phenotype was determined using a LRT to evaluate whether gene expression was statistically indistinguishable from WT. As expected, chimeras with DBD from species more closely related to *S*. *cerevisiae* (Clade 1–2) were better able to recapitulate wild type gene expression (Fig 4). *K*. *lactis* and *C*. *albicans* Mbp1BDs could recapitulate the expression of 26 and 27 (38.5%) members of the SBF-activated regulon, respectively (19 shared between *K*. *lactis* and *C*. *albicans* of the original 68-gene regulon). On the other hand, *N*. *crassa* and *S*. *pombe* ResBDs recapitulated wild type expression of 10 and 8 genes from our SBF-activated regulon, respectively. Although most of the SBF-activated regulon was rescued by the most closely related DBD (Fig 4), 48.5% of the SBF-activated regulon (33 out of 68 genes) was not rescued by any of the DBD swap experiments, including many of the most strongly regulated genes (Fig 4; *S*. *cerevisiae*-specific SBF regulon). Only three genes from our SBF-activated regulon were rescued by all the DBDs used in our experiments (*DSE2*, *CIT2*, *TRF5*). Using real time PCR analysis we validated changes in expression of four target genes, *SVS1*, *CLN1*, *CLN2* and *PCL1*, in eight chimeras containing Swi4 AD and detected a gradual decrease in their periodic expression in correlation with their evolutionary distance, RNA-seq data and phenotypes when tested on the background of *mbp1Δ* (S9 Fig).

### Promoter analyses suggest an MCB-like ancestral motif for the G1/S regulon

**Fig 5:**
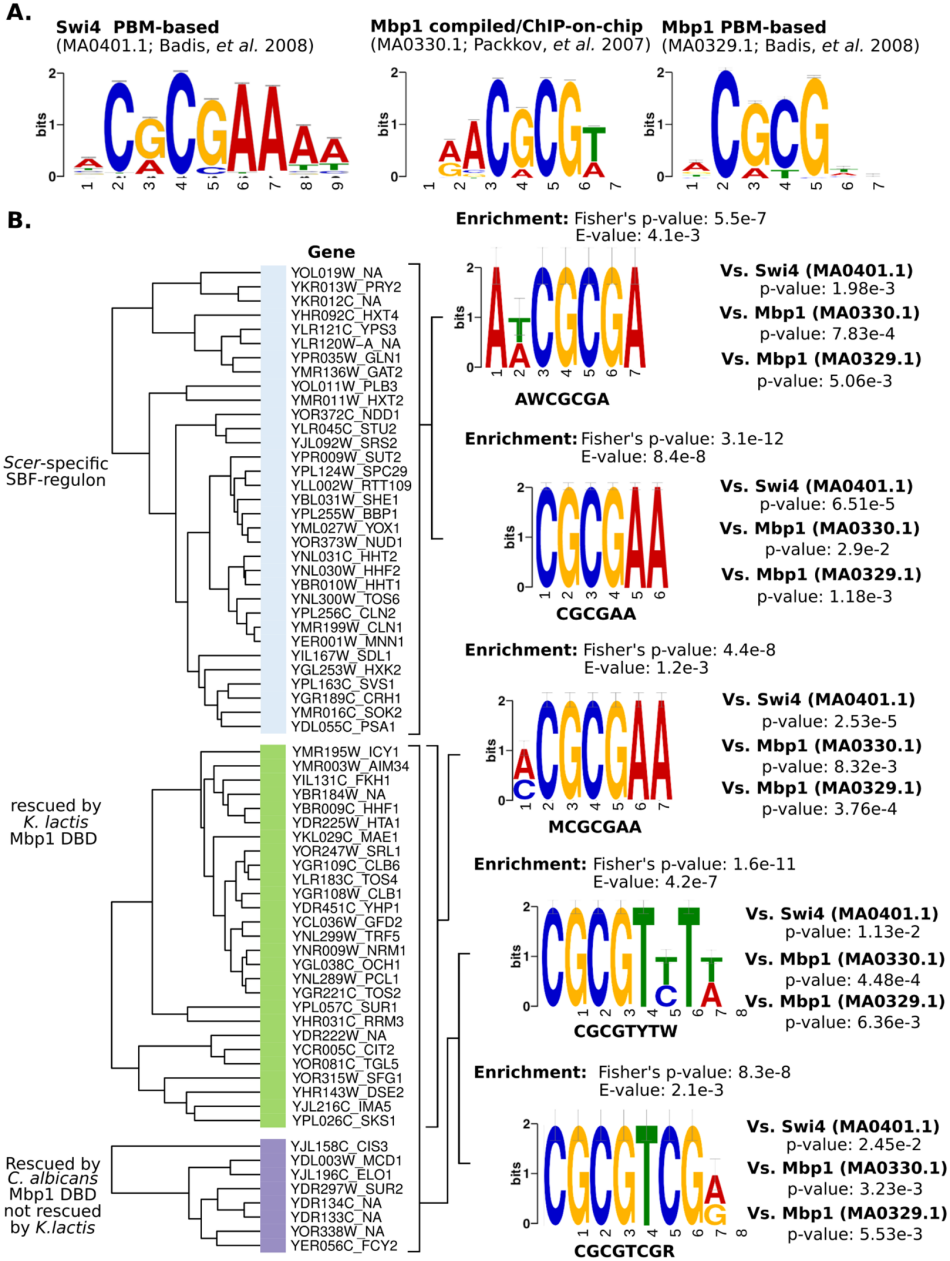
Regulon-specific promoter motif enrichment analysis. (A) Known DNA-binding motifs for Swi4 and Mbp1 [24]. (B) Motif enrichment analyses relative to non-significant differentially expressed genes was performed with MEME-ChIP [25] on the 1000bp upstream regions of the genes from each of the rescued SBF-regulons and compared to known motifs for Swi4 and Mbp1 (JASPAR ID shown). Cumulative rescue plot shows *Scer*-specific genes in clear blue (not rescued by any chimera) followed by genes rescued by *K*.*lactis* Mbp1 DBD (green). *C*.*albicans* Mbp1 rescued regulon would be the subset in purple plus most of the genes previously rescued by *K*.*lactis* Mbp1 DBD (green) (see Fig 4).

Current consensus and *in vitro* protein binding microarray data indicate that *Sc*Swi4 binds the motif CRCGAAA while *Sc*Mbp1 binds ACGCGT [4] (Fig 5A). To determine if there were differences in the promoter regions of members of the SBF-activated regulon that might explain why some genes were rescued by some chimeric TFs and not others, we performed motif enrichment analyses on the 1000 bp region upstream of the transcription start site for each of our chimeric TFs (*S*. *cerevisiae*-specific regulon, *K*. *lactis* Mbp1-rescued, and *C*. *albicans* Mbp1-rescued, Fig 4). We were unable to do this analysis for *N*. *crassa* and *S*. *pombe* because there were too few Res-rescued promoters. We found differences in the enriched motifs from each of the subsets of the SBF-activated regulon (Fig 5B), with a tendency for MCB-like motifs in the *C*. *albicans* Mbp1-rescued regulon (CGCGT[T/C]T[T/A]). As expected, the motif with the highest enrichment in the *Scer*-specific regulon corresponds to a Swi4 motif (Fig 5; CGCGAA: p-value 3.1e-12). Although we expected only an enrichment for a “pure” SCB sites in the *S*. *cerevisiae*-specific regulon, we also detected enrichment for a second motif more MCB-like than SCB-like (p-value 7.83e-4 vs. 1.98e-3, respectively), possibly due to the 5’ “A[A/T]”. *K*. *lactis* Mbp1-rescued regulon enriched motif ([A/C]CGCGAA) shows higher similarity of *S*. *cerevisiae* Swi4 (p-value 2.53e-5) than to Mbp1 (p-value 8.32e-3), but this difference is subtle when compared with the PBM-based Mbp1 motif (MA0329.1; p-value 3.76e-4). Interestingly, the motifs enriched in *C*. *albicans* Mbp1-rescued regulon have a tendency towards more MCB-like motifs by replacement of the Swi4 “A” by a “T” (i.e. CGCGA to CGCGT). This Mbp1-like motif would be consistent with a Res-like ancestor that binds expanded SCB/MCB-like motif (RCB), as suggested by current motifs found in *S*. *pombe*.

### Swi4 binds SCB motifs with much higher affinity than MCB-like ancestral motifs

**Fig 6:**
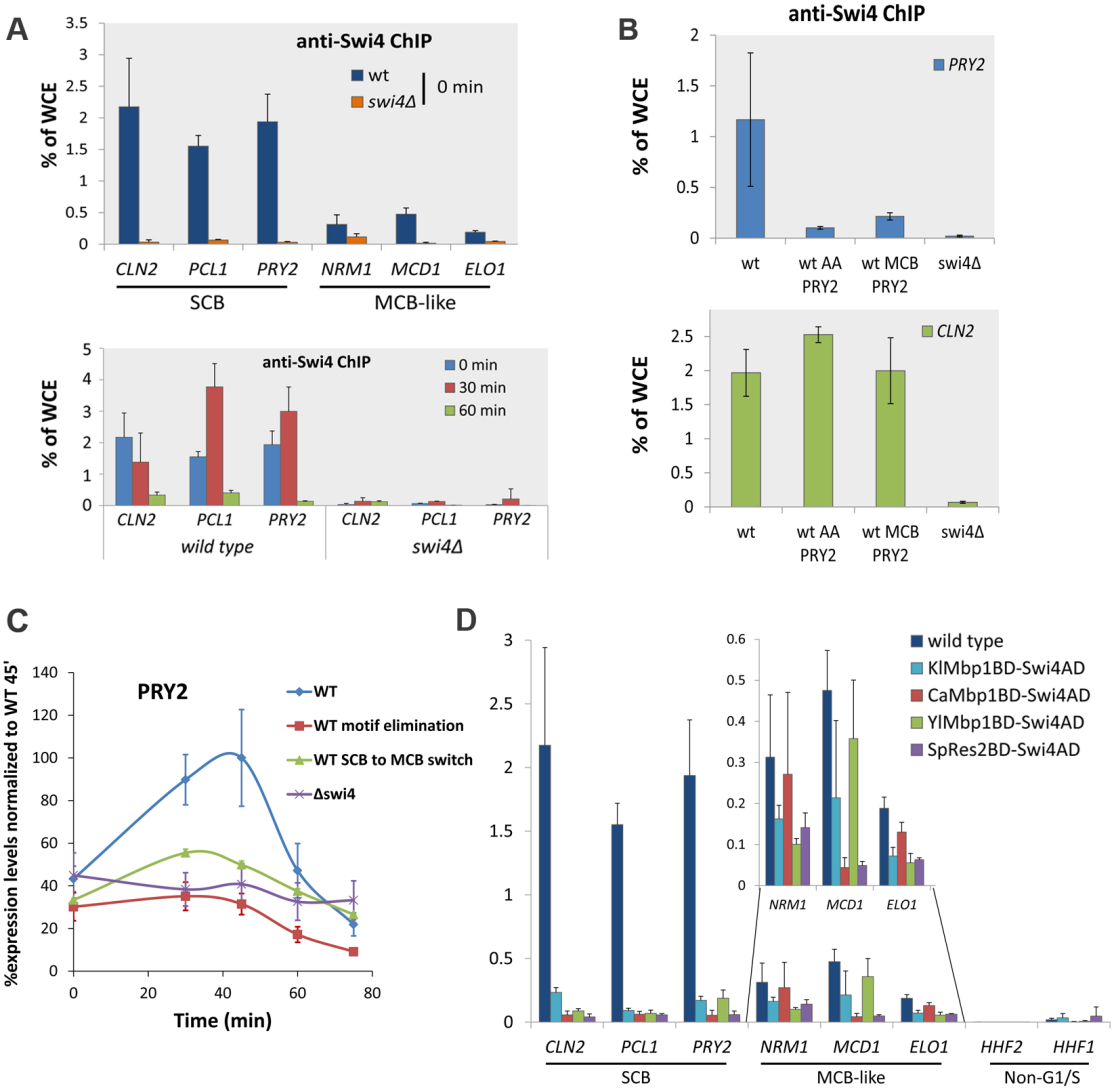
Chimeric Swi4 TFs bind MCB-like ancestral motifs, but not SCBs, which are required for binding and regulation by wt Swi4. (A-B) anti-swi4 Chromatin Immunoprecipitation (ChIP). Binding to *CLN2*, *PCL1* and *PRY2*, SCB motifs containing promoters (SCB), and *NRM1*, *MCD1*, and *ELO1*, MCB-like motifs containing promoters (MCB-like) was analyzed by ChIP. Binding was assessed in alpha factor arrested cultures (0 min in top panel) and arrested (0 min) and released cultures (30 min and 60 min, bottom panel). Enrichment is indicated as percentage of input of Whole Cell Extract (% of WCE). ChIP analysis was carried out in (A-B) wild type (wt) and *swi4*Δ cells and wild type cells harboring mutations in the *PRY2* promoter disrupting the core SCB motif (wt AA *PRY2*) and changing it to MCB-like motifs (wt MCB *PRY2*). (C) Analysis of *PRY2* expression in wild type (wt), *swi4*Δ and *PRY2* promoter mutants (AA and MCB-like). (D) ChIP analysis of wild type (wt) and all chimeric TFs used in the RNA seq experiment, *K*. *lactis* (*Kl*Mbp1BD-Swi4AD), *C*. *albicans* (*Ca*Mbp1BD-Swi4AD), *N*. *crassa* (*Nc*ResBD-Swi4AD) and *S*. *pombe* chimeras (*Sp*Mbp1BD-Swi4AD) for indicated promoters.

Our results suggest that some SBF target genes, which can be regulated by closely related DBDs, are enriched for MCB-like motifs in their promoter. To test the ability of Swi4 and chimeric TFs with Swi4AD variants to bind SBF target promoters with different motifs we carried out anti-Swi4 Chromatin ImmunoPrecipitation (ChIP). Specific polyclonal antisera to the C-terminal domain of Swi4 [26], recognizing the common Swi4AD in WT and chimeras, was used for all ChIP experiments. We analyzed a set of SCB/MCB-like targets including the MCB targets *NRM1*, *MCD1*, and *ELO1* and SCB targets *CLN2*, *PCL1* and *PRY2*, representing SBF target genes. Since Swi4 binds SBF target promoters only during G1, we measured binding affinity of Swi4 for these promoters in alpha factor arrested cells using *swi4*Δ cells as a negative control. Our data shows that, as expected, Swi4 ChIP specifically enriches SBF target promoter sequences (Fig 6A). Unexpectedly, we find that Swi4 binds SBF target promoters with SCB motifs with much higher affinity than those with the MCB-like motifs. To further establish that the high affinity to promoters with SCB motifs is specific, we carried out Swi4 ChIP on cell cycle synchronized cells (Fig 6A bottom panel). These data show that binding is lost once cells enter S phase at 60 minutes after alpha factor release, confirming Swi4’s binding specificity for these promoters. Whilst it has been known for some time that SBF binds target promoters with different affinity, the basis of this has remained unclear. Our data indicate that Swi4’s affinity for the MCB-like motifs, which likely resemble the ancestral motifs, is sub-optimal compared to the clade 1 specific SCB motif.

### SCB motifs allow high affinity Swi4 binding and regulation of the SBF target *PRY2*

To test if SCB motifs are required for Swi4’s ability to bind with high affinity and regulate transcription we carried out ChIP and expression analysis on two *PRY2* SCB motif mutants (Fig 6). We mutated the SCBs at the *PRY2* endogenous promoter to resemble MCB-like motifs (ATCGCGA to AACGCGT: wt MCB) and disrupted the core binding motif (CGCG to CAAG: wt AA *PRY2*) to validate that expression is controlled by the SCB motifs. Anti-Swi4 ChIP analysis was carried out to establish binding affinity to the *PRY2* promoter in the *PRY2* promoter mutants (wt MCB *PRY2* and wt AA *PRY2*) and wt and *swi4*Δ cells as positive and negative controls, respectively while *CLN2* was used as a positive control (Fig 6B). Whereas Swi4 binds the *CLN2* promoter with similar affinity in the wt and *PRY2* promoter mutant strains, it is unable to bind the *PRY2* promoter when the core sequence is mutated and only binds with low affinity when the SCBs are mutated into MCB-like motifs (Fig 6B). These data show that the SCB motifs are required for high affinity binding of Swi4 to the *PRY2* promoter. To establish the importance of binding affinity on the ability of Swi4 to regulate *PRY2* expression, we carried out expression analysis in alpha factor arrested and released synchronized cells at 0, 30 and 60 minutes in these same strains (Fig 6C). As expected, mutating the core motif (AA) resulted in loss of cell cycle periodicity with expression levels comparable to *swi4*Δ. This demonstrates that the SCB motifs are required for SBF-dependent regulation. Importantly, whilst not to the same extent, mutating the SCBs into MCB-like motifs severely reduce *PRY2* expression levels indicating that the SCB motifs in the PRY2 promoter are required for Swi4-dependent regulation.

### MCB-like motifs in SBF-regulated genes allow their binding by chimeric TFs

Our data indicates that the clade 1 specific SCB motif, not present in distantly related yeast, represents an optimized DNA binding motif solution that is required for proper transcriptional regulation by Swi4. In addition, we find that chimeric TF variants with Swi4AD can regulate SBF target genes that are enriched for MCB-like motifs in their promoter, not SCBs. Based on this observation, we predicted that chimeric TFs can bind the promoters of genes with the MCB-like motifs, but not those with SCBs. To test this, we carried out anti-Swi4 ChIP on WT and the *K*. *lactis*, *C*. *albicans*, *N*. *crassa* and *S*. *pombe* chimeras (Fig 6D). Again, we analyzed the set of SCB/MCB-like targets (*NRM1*, *MCD1*, and *ELO1*) and SCB targets (*CLN2*, *PCL1* and *PRY2*). These data show that the chimeras bind the promoters of SBF target genes with MCB-like motifs to the same extent as WT Swi4. However, the chimeras do not bind promoters with SCBs to the same extent as WT Swi4. These results indicate that the chimeras’ capability to regulate only a subset of SBF target genes is likely based on their ability to bind the MCB-like motif, but not SCBs.

## Discussion

Our results point to a mechanism of G1/S transcription network expansion that is characterized by gene duplication and gradual co-evolution of the DBD and its promoters. By utilizing different chimera TFs, we have shown that homologous MBF/SBF DBDs of yeast representative clades II-V species when fused to Swi4 AD can regulate a subset of the SBF regulon. This suggests that the set of G1/S target genes regulated by SBF likely represents the ancestral G1/S transcriptional regulatory network. In contrast, we found that the homologous DBDs when fused to Mbp1 AD are unable to rescue the regulation of MBF dependent genes, which suggests that functional specificity of Mbp1 evolved more recently during yeast evolution. Our phenotypic and expression analysis shows that DBDs from more closely related yeasts fused to Swi4 AD can better recapitulate wild type expression of SBF-dependent genes than those from more distantly related yeasts (Figs 3 and 4). Interestingly, our data reveals that the promoter sequences of the SBF targets regulated by the orthologues DBDs tend to contain more MCB-like motifs consistent with a Res-like ancestor, as found in *S*. *pombe* (Fig 5). We show that homologous DBDs can bind to SBF target promoters with MCB-like motifs, but not those with SCBs, for which the wild type Swi4 has a high affinity.

### MCB vs SCB

In budding yeast the MBF transcriptional complex recognizes the conserved MCB element (*ACGCGT*) whilst the SBF complex is thought to bind the SCB recognition motif (*CRCGAAA*). Our findings show that a subset of SBF-dependent targets contain a MCB-like sequence demonstrating that Swi4 BDs can recognize both MCB and SCB regulatory sequences across species. These results suggest that Swi4/SBF likely represents the ancestral G1/S transcriptional regulator that activates the transcription of genes containing both the modern SCB motif and that the ancestral binding sequence was MCB-like. This is in line with previous functional analysis of Mbp1 and Swi4 in *C*. *albicans* that identified Swi4 as the functionally important TF recognizing MCBs ([27]). Further support for the binding flexibility of MBF and SBF comes from studies performed with plasmids, containing repeated MCB or SCB sequences, that show no significant bias of MBF or SBF towards binding either sequence [15]. In accordance, the core *CGCG* recognition sequence is highly conserved and can be found in G1/S target promoters from yeast to human [3]. Thus, it seems that while the core-conserved sequence (*CGCG*) might be important for binding, the specificity is likely dictated by much larger stretches of DNA sequence gene and the TF binding site. In addition, biochemical and structural analysis of Mbp1/Swi4 DBD revealed that residues that are essential for DNA binding are highly conserved suggesting a conserved mode of protein-DNA interaction [11, 15, 16]. Thus, tuning of specificity in these DBDs during evolution is probably achieved through a series of subtle non-conserved substitutions enabling binding to a wider range of promoters to enable network expansion. Our work also suggests a possible important role for the AD in dictating the specificity of gene regulation.

### SBF vs MBF

Although the dynamic expression patterns of SBF and MBF-dependent targets appear similar in an unperturbed division cycle, the mechanisms of regulation are different. Remarkably, while SBF is a transcriptional activator required to activate G1/S transcription during G1, MBF is a transcriptional repressor that inhibits transcription outside of G1 [9]. Thus, inactivation of SBF inhibits the expression of its targets, while inactivation of MBF leads to constitutively high levels of MBF-dependent transcription. Our work identifies the SBF activator complex as the likely ancestral G1/S transcriptional regulator. This is in line with work carried out in *S*. *pombe* and *C*. *albicans* that shows that activation, rather than repression, of G1/S transcription is essential for cell viability [28, 29].

Overall, our results suggest that the G1/S network expansion in *Sc* could have co-occurred through the divergence of the ancestral RCB into a Res-like MCB and a more divergent SCB in the regulatory regions of the core G1/S regulon and in new genes, thus expanding the existing/ancestral SBF regulon.

## Materials and methods

### Phylogenetic analysis

Homologs from the SBF/MBF family within Dikarya were retrieved as previously described [5]. The phylogenetic analysis was performed on an alignment build using MAFFT-L-INS-i (-maxiterate 1000) [30, 31] We then used probabilistic alignment masking using ZORRO to create different datasets with varying score thresholds, where the top 20% scoring columns was chosen as the best alignment (S1 Dataset). Next, we used ProtTest 3 to determine the empirical amino-acid evolutionary model that best fit each of our protein datasets using several criteria: Akaike Information Criterion, corrected Akaike Information Criterion, Bayesian Information Criterion and Decision Theory [32]. Last, for each dataset and its best-fitting model, we ran different phylogenetic programs that use maximum-likelihood methods with different algorithmic approximations (RAxML and PhyML) to reconstruct the phylogenetic relationships between proteins. For RAxML analyses, the best likelihood tree was obtained from five independent maximum likelihood runs started from randomized parsimony trees using the empirical evolutionary model provided by ProtTest. We assessed branch support via rapid bootstrapping (RBS) with 100 pseudo-replicates. PhyML 3.0 phylogenetic trees were obtained from five independent randomized starting neighbor-joining trees (RAND) using the best topology from both NNI and SPR moves. Non-parametric Shimodaira-Hasegawa-like approximate likelihood ratio tests (SH-aLRTs) and parametric *à la Bayes* aLRTs (aBayes) were calculated to determine branch support from two independent PhyML 3.0 runs.

### Plasmids and yeast strains

In order to generate chimeric TFs expressed from its native promotor, we first constructed a set of plasmids. For the generation of plasmids containing Mbp1 and Swi4 genes each gene was amplified by PCR from *S*. *cerevisiae* in two parts: the first one is the promoter and the second one is the ORF gene and its terminator. The PCR amplification products of the whole gene of Mbp1 or Swi4 were introduced into pRS315 centromeric plasmid that was cut using BamHI and SalI or NotI and SalI sites, respectively by homologous recombination into yeast (W303 strain). The resulting pRS315-Mbp1-terminator and pRS315-Swi4-term that were cut using SacI and NotI and the promoter PCR amplification product was added. The result of this homologous recombination in yeast (W303) led to the creation of pRS315-pro-Mbp1-term and pRS315-pro-Swi4-term. A similar process was performed in order to create the pRS316 plasmid where the whole gene with its promoter and terminator were amplified by PCR and introduced to the digested plasmid by SalI and SacI by homologous recombination in yeast (W303). The chimeric Mbp1 TFs were constructed by amplification of the DBD of the TF from: *K*. *lactis*, *K*. *waltii*, *C*. *albicans*, *D*.*hansenii*, *Y*.*lipolytica*, *N*.*crassa* and from *S*. *pombe* (RES1 DBD and RES2 DBD). The DBDs PCR products were transformed with the Mbp1 activation domain (AD) into yeast (W303) to generate the pRS315-pro-chimericMbp1-term plasmid. The same process was done with Swi4 AD to generate chimeric Swi4 TFs shown in Fig 2. For a complete strain list please see S2 Table.

### Viability assay

In order to check the effect of the chimeric TFs, we generated *swi4Δmbp1Δ* strain complemented with pRS316 plasmids expressing Mbp1 or Swi4. The *swi4Δmbp1Δ* was generated on the background of 15Daub containing the *swi4Δ* (genotype: *ade1*, *leu2-3*, *112 his2 trp1-1 ura3 swi4*: *KanMX Δbar1*). First the 15Daub containing the *swi4Δ* was transformed with the pRS316-pro-Swi4-term and plated on SC-ura. Then, *mbp1Δ* was generated by transformation of CloNAT cassette PCR amplification product followed by platting on SC-ura+G418+nat. The *swi4Δmbp1Δ* strain was then transformed with different pRS315 plasmids containing the different chimeric TFs and plated onto SC-leu-ura. To examine the effect of the chimeric TFs as a sole source of Mbp1/Swi4 in the yeast, the colonies were replicated to SC-leu+5FOA plates and incubated two days in 30°C (S2 Fig).

### Generation of genomic integrated strains

To integrate the different chimeric Swi4 TF genes into the genome, we used the previously used strain (15Daub *swi4Δ*) containing the KanMX cassette. To replace the KanMX with the chimeric TFs we used the SB221 plasmid as previously described [33]. Briefly, the whole chimeric Swi4 TFs were amplified with a 500bp flanking of the promoter and transformed into 15Daub *swi4Δ* together with a PCR product amplified from the SB221 to include the URA3 gene flanked by KanMX recognition site. This allowed for homologous recombination into the strain at the endogenous *SWI4* locus. After the integration of the TF chimeras *MBP1* deletion to CloNAT by homologous recombination in those strains were performed by the PCR amplification product of the CloNAT targeted to the promoter and terminator of *MBP1*. In order to generate yeast strains containing mutations within their promoters we deleted 1000bp upstream to the gene. Specifically, the yeast strains were transformed with PCR amplification of the CloNAT gene that by homologous recombination replaced the endogenous promoter in the genome, and the cell were plated on YPD+CloNAT. The mutated promoters were generated using the CRISPR/Cas9 system with a specific guide to the CloNAT gene and donor DNA that contained the promoter with the specific mutations flanked by regions enabling genomic integration following CAS9 cleavage.

### Alpha factor synchronization

Single colonies of the strains containing the WT or the chimeric TFs were grown over-night in 5 ml YPD at 30°C, then the cultures were diluted to OD_600_ ≈ 0.1 in 15 ml YPD and grew at 30°C to OD_600_ ≈ 0.4 (logarithmic stage). Next, 100ng/ml of alpha factor was added and the cultures were grown at 30°C for another two hours [34]. Synchronization was checked by microscope (unbudded shmoo shape). Synchronized cells were washed two times in fresh YPD to get rid of the alpha factor and continued to grow in fresh YPD media.

### Growth curves analysis and temperature sensitivity assay

Cells were grown in 7 ml of SC over-night at 30°C. Next morning, cells were diluted to OD_600_ ≈ 0.1 in fresh SC medium and grew for 24 hours in 96-well plate (Nunc). The plates were constantly shaken in Varioscan Flash plate reader (Thermo Scientific) at 30°C and OD measurements (OD_600_) were taken every 30 minutes. The resulting curves were used to calculate the generation times (τ) of the different strains using the OD_t_ = OD_0_∙2^t/τ^ equation. Logarithmic transformation allowed obtaining the linear equation Log_2_OD_t_ = Log_2_OD_0_+t/τ, where the slope value of the linear fit is 1/τ. The curves are averages of at least three independent repeats. The curves include the exponential (logarithmic) growth phase allowing the calculation of the generation times (τ) of the different strains as described above. For temperature sensitivity the genomic integrated strains with the chimeric Swi4 TF and *mbp1Δ* were grown over-night at 30°C in 7ml of liquid SC medium, washed twice and diluted to an initial OD_600_ ≈ 0.6. A series of serial dilutions was conducted and the cells were spotted on SC plates. The plates were incubated in 30°C and 37°C [35].

### Cell imaging

Cells were grown in YPD liquid medium at 30°C to OD_600_ ≈ 0.6 and then photographed using Zeiss Axioplan 2 microscope and QImaging QIClick CCD camera. In order to explore morphological differences between the strains, we grew each of the genomic integrated strain with the *mbp1*Δ and divided them to a 96 well plate (Nunc) suitable for microscopy analysis and used the Operetta High Content Imaging System (Perkin Elmer). Bundles of cells were defined as two or more cells connected to form a large cell size and elongated cells were defined to have a bud size that is higher than 3 microns.

### Western blot analysis

Exponentially growing cells were diluted to the same OD_600_ in 50 ml, centrifuged and cell lysates from the WT and chimeric TFs were prepared for Western blotting. We used alkaline extraction by 5-minute room temperature incubation of the pellet in 100 mM NaOH solution prior to resuspension and boiling for 3 minutes in SDS sample buffer. Approximately 10 μg/μl of total proteins were loaded onto 12% SDS-PAGE and analyzed by Western blot analysis using primary antibodies anti-Mbp1 or anti-Swi4, (1:2000) followed by α-rabbit HRP-conjugated secondary antibodies (1:3000) [26].

### Anti-Swi4 ChIP analysis

For each immuno-precipitation, exponentially growing cells were diluted to the same OD_600_ in 50 ml. In order to cross-link DNA and protein we used 1.25 ml formaldehyde (37%) and incubate it for 20 minutes at room temperature. Then 2.3 ml of 2.5 M glycine was added to stop the reaction and incubated for another 5 minutes. Next, the pellet was washed twice in TBS and frozen in liquid nitrogen. Frozen pellets were mechanically disrupted by 30 minutes vortexing with glass beads (BioSpec) in lysis buffer (50 mM Tris-HCl pH 7.5, 1% Triton X-100, 250 mM NaCl) containing protease inhibitors (Complete Mini, Roche) and phosphatase inhibitors (Phosphatase Inhibitor Cocktail 1, Sigma-Aldrich) at 4°C. Lysates were spun for 5 minutes at top speed and supernatant sonicated (QSonica Q800R sonicator; amplitude 100%, process time 5 min with pulse-on 30 sec and pulse-off 2 min) and then immunoprecipitated with anti-Swi4 polyclonal sera by incubating lysates overnight at 4°C then 35 μl of protein A Sepharose beads were added for additional 3 hours at 4°C. Beads were washed (straight after 3 hours of incubation), spun down and DNA fragments were purified with 10% Chelex Resin solution (Bio-Rad) and boiling for 10 min [36]. Quantitative PCR (qPCR) was performed on specific target genes using the iQ SYBR Green supermix (Bio-Rad) kit. Samples were run on a Chromo-4 Real-Time PCR Detector (Bio-Rad). Data was analysed using MJ Opticon Analysis Software 3.0 [37].

### RNA extraction and reverse transcriptase qPCR analysis

Cells were synchronized by alpha factor arrest and release (see above) and 1.5 ml cells were collected at different time points (0, 30 minutes) and frozen in liquid nitrogen. Next, total RNA extraction was performed using Epicentere kit. Each RNA sample was used as a template for synthesis of single strand cDNA for detection of different target gene of the TF by reverse transcriptase (Thermo) using oligo-dT as primer. Relative transcript levels were then determined by qPCR analysis using a 7300 Applied Biosystem machine. The reaction mix contained 10 μl of SYBR green mix (Applied Biosystem), 10 μM of primers, 2 ng/μl cDNA and DDW to total reaction volume of 20 μl. *ACT1* was used as the internal standard. The resulting curves represent the averages of at least three independent repeats.

### RNA-seq, differential gene expression and GO analyses

Total RNA was extracted from alpha factor synchronized WT and *swi4Δ* strains, as described in qPCR, and checked for quality using agarose gel or Bioanalyzer. The libraries were constructed using the Tru-seq RNA library kit (Illumina) and checked by Bioanalyzer and Quabit for quality assessment. The samples were run on the Illumina HiSeq 2500 instrument at the Technion Genome center (Technion, Israel). Original complete dataset can be found in Dryad Digital Repository (doi: 10.5061/dryad.2rf10). The quality of the raw sequence data was assessed using FastQC (http://www.bioinformatics.babraham.ac.uk/projects/fastqc/). Reference genome and annotations were downloaded from Ensembl ftp, fasta and GTF annotation file (ftp://ftp.ensembl.org/pub/release-75/gtf/saccharomyces_cerevisiae/Saccharomyces_cerevisiae.R64-1-1.75.gtf.gz). Reads were aligned to reference genome using STAR [38] and Htseq-count was used for counting number of mapped reads per gene (http://www-huber.embl.de/users/anders/HTSeq/doc/overview.html). All differential expression analyses were performed on S2 Dataset using DESeq2 [39]. We defined the SBF regulon as the set of genes that show significant changes in *swi4*Δ relative to wild type over time (0, 30 min). These genes were detected using likelihood ratio test (LRT), in which we compared the full model (~strain + time + strain:time) with a reduced model in which we removed strain specific differences over time (the interaction term strain:time). Genes that show differences in their pattern of expression at a 10% false discovery rate (i.e. Benjamini-Hochberg adjusted p-value of 0.1) were considered significant. Genes that change their pattern of expression in the same way in the wild type and *swi4Δ* strain are not considered significant. We then ranked significant genes by their strength of regulation (overexpressed (TF-repressed) or negatively regulated (TF-activated)) based on log2 fold change in expression relative to WT. Heatmaps were generated with the “pheatmap” R package from rlog-transformed data, while the differential expression analyses were performed on raw data. The regularized-logarithm transformation (rlog) addresses the problem of RNA-seq data in which the variance increases with its mean. With the rlog, genes with low counts are shrunken towards gene’s averages across samples. The rlog-transform is not different from a log2 transform for genes with high counts. Gene ontology analyses were performed for the “Biological Process” ontology using the clusterProfiler R package [40] using as universe all genes represented in our RNA-Seq dataset. Significance was assessed using Benjamini-Hochberg corrected p-value threshold of 0.01 and q-value cutoff of 0.05.

### Motif enrichment analyses

Enriched motifs in each of the TF-activated rescued subsets were estimated with MEME-ChIP program through the MEME Suite v4.11.2 [25]. Enrichment (p-value < 0.05) was calculated for the promoter regions (comprising 1000 nucleotides upstream of the transcription start site) of the target genes relative to the promoter regions of genes (5565 genes) that showed no significant differential expression between *swi4*Δ and wild type through the likelihood ratio test (discriminative mode). Retrieved SCB and MCB motifs (DREME) were then compared to the JASPAR CORE (2016) database for Fungal transcription factors (Tomtom) to determine how similar they were to the motifs of budding yeast Swi4 and Mbp1 (p-values in Fig 5). To reconstruct the MCB1 and MCB2 motifs from *S*. *pombe* in Fig 1, the promoter regions (1000bp upstream) for the set of genes previously suggested to contain MCB1 and/or MCB2 motifs [6] were analyzed with MEME to identify enriched motifs. We recovered an MCB2 motif with an E-value 5.4e-27, but were unable to recover the MCB1 motif. The published MCB1 motif was reconstructed by scanning for enrichment (p-value < 0.001) of the corresponding seed motif (MCB1: AACGCGTT from[6]) in the promoter regions (1000bp upstream) for the set of genes previously suggested to contain MCB1 and/or MCB2 motifs [6] using the FIMO package from the MEME suite Suite v4.11.2[25]. Similarly, we reconstructed the published MCB motif for *C*. *albicans* by scanning for enrichment (p-value < 0.001) of the corresponding seed motif (MCB: ACGCGW) in the promoter regions (1000bp upstream) from genes from the G1/S regulon [4] using the FIMO package.

## Supporting information

S1 Fig Computational prediction of unstructured regions in Mbp1 (A) and Swi4 (B) using the IUPred web server. The predictor score for disorder tendency is plotted against the residue number. Residues with a higher and lower score than threshold (0.5) are considered to be in disordered and ordered regions, respectively. Large regions of the TFs are predicted to be disordered, but known functional domains including the DBD, Ankyrin repeat and the C-terminal domain are predicted to be ordered. This analysis supports a ball-and-string model where functional ordered regions are connected by flexible disordered regions. The vertical black line highlights the position of recombination for the generation of chimeric TFs. In the case of Mbp1 this is position 125 and in Swi4 this is position 165. The functional domains are annotated according to Siegmund and Nasmyth [20].
(TIF)

S2 Fig The plasmid shuffling approach. To examine the contribution of the chimeric TFs to *S*. *cerevisiae* viability we used a *mbp1Δswi4Δ* strain that was complemented by the WT Swi4 expressed from a URA3 centromeric plasmid and the chimeric TF expressed from a LEU2 centromeric plasmid. Upon replication of colonies on plates containing 5FOA the URA3 plasmid containing the WT Swi4 gene is lost and the plasmid encoding the chimeric TF is the sole source of Swi4 or Mbp1 expression in the cell. In cases where the chimeric TF can complement the deletions, the yeast will be viable and will grow on 5FOA plates (see Fig 2).
(TIF)

S3 Fig Western blot analysis of chimeric TFs with Mbp1AD and Swi4AD. (A) Expression levels of chimeric TFs with Mbp1AD in *S*. *cerevisiae*, as assessed using α-Mbp1 antibodies. (B) Expression levels of chimeric TFs with Swi4AD in *S*. *cerevisiae* assessed using α-Swi4. The expression of PSTAIR (Cdc28p) was monitored as a loading control using α-PSTAIR antibodies.
(TIF)

S4 Fig Expression analysis of Mbp1 and Swi4 target genes in strains containing chimeric TFs with Mbp1AD by qPCR. (A) Analysis of MBF target gene-*CDC21* and (B) MBF and SBF target gene- *TOS4* shows that only clade 1 chimeric TFs exhibit periodic gene expression. (C) SBF target gene-*SVS1* analysis shows non periodic expression in strains expressing chimeric Mbp1 containing DBDs from representative strains of clades 2–5.
(TIF)

S5 Fig In vivo phenotypic analysis of chimeric TFs with Swi4AD. (A) Temperature sensitivity analysis of chimeric TFs with Swi4AD integrated into the *S*. *cerevisiae* genome of *mbp1*Δ strains. *Kl*Mbp1BD-Swi4AD, *Kl*Swi4BD-Swi4AD, *Ca*Mbp1BD-Swi4AD and *Sp*Res1BD-Swi4AD strains are not sensitive to restrictive temperatures (37°C), while *Nc*ResBD-Swi4AD and *Sp*Res2BD-Swi4AD are viable only at optimal conditions (30°C). (B) Growth analysis of chimeric TFs integrated into the genome in *mbp1Δ* background. The strains contain *Kl*Mbp1BD-Swi4AD, *Kl*Swi4BD-Swi4AD and *Ca*Mbp1BD-Swi4AD (Green, light green and light blue lines, respectively) display similar to the WT and *mbp1*Δ strain growth rate (Grey line and the dark grey line, respectively). Strains expressing the chimeric *Yl*ResBD-Swi4AD (pink line), *Nc*ResBD-Swi4AD (brown line), *Sp*Res1BD-Swi4AD (light brown line) and *Sp*Res2BD-Swi4AD (yellow line) exhibit decreased growth rate. (C) Quantification of the cell morphology of all strains expressing chimeric TFs as analyzed by light microscopy.
(TIF)

S6 Fig Cell cycle synchrony and G1/S transcript levels in wild type, *swi4*Δ and chimeric TFs with Swi4AD. Cultures of indicated strains were synchronized by alpha factor arrest and released at time 0. Top, relative mRNA levels of *RNR1* and middle, relative mRNA levels of *CLN2* were analyzed by q-PCR during the cell cycle. Light red bar indicates 'SBF-dependent' transcription levels (difference between wild type and *swi4*Δ). Expression levels are plotted as percentage of highest value detected in wild type experiment (100%). Bottom, budding index (% budded cells) is provided as an indicator of cell cycle progression after release from alpha factor arrest.
(TIF)

S7 Fig SBF-regulated genes and their rescue by evolutionarily related DNA-binding domains. Gene expression profile of SBF-regulated genes at two time points (0 and 30 mins) after release from alpha-factor block. We plot the regularized-logarithm transformed data of each gene (row) for each strain (wild-type or DBD chimeras, columns) for visualization. All RNA-seq experiments were done in triplicate. The RNA-seq expression of each gene (heatmap) is plotted as a Z-score relative to the mean and standard deviation of all values on the same row. Statistical analysis with DESeq2 was performed on the raw data. SBF-activated genes (68 genes) and SBF-repressed genes (64 genes) are shown in red and black, respectively. Genes that had no statistical difference between RNA-seq expression at 0 and 30 mins between wild-type and chimeras were grouped together within the same contiguous box. Genes (rows) outside the box indicate that expression was significantly different from wild-type and not rescued by DBD chimeras of the corresponding species (column). Genes were organized top-down starting with the genes from SBF-regulated genes that were only rescued by *Sc*Swi4BD (Scer-specific SBF regulon). We then list genes from SBF-regulated genes that were also rescued by *Kl*Mbp1 (*Klac* Mbp1BD-rescued regulon). Genes rescued by *Kl*Mbp1BD were usually rescued by *Ca*Mbp1BD with occasional rescue by *Nc*ResBD and *Sp*Res2BD. We then show those genes not rescued by *Kl*Mbp1BD, but which were rescued by *Ca*Mbp1BD with occasional rescue by *Nc*ResBD and *Sp*Res2BD. Last, we show those genes not rescued by *Kl*Mbp1BD, *Ca*Mbp1BD, *Nc*ResBD, but which were rescued *Sp*Res2BD. Each module was organized using hierarchical clustering of RNA-seq profiles.
(TIF)

S8 Fig Genes involved in cell cycle processes are enriched in our SBF-activated regulon. GO enrichment analysis for the SBF-activated regulon (68 genes) in “biological process” ontology terms were determined using p_BH_-value threshold of 0.01 and q-value threshold of 0.05. Our null model was all measured genes in our RNA-seq experiment. Length of the bar represents genes with the corresponding ontology term (e.g. 24 genes have “cell cycle” GO term), color of the bar represents enrichment p_BH_-value (also shown in bar). The same gene can belong to multiple GO categories. There was a total of 14 categories enriched in this dataset, but we only show the top 10.
(TIF)

S9 Fig Expression of Swi4 target genes in strains containing different chimeric TFs with Swi4AD. Relative expression of Swi4 target genes *CLN1*, *CLN2*, *PCL1* and *SVS1* was determined by qPCR analysis. (A) *CLN1* expression levels in the strains containing chimeric TFs show reduced expression in more distantly related species. In strains containing *Kl*Swi4BD, *Kl*Mbp1BD and *Ca*Mbp1BD, the gene expression of *CLN1* is periodic, although peak levels are lower than in WT. Other strains with chimeric TFs show no periodic expression which is similar to the *swi4*Δ strain. (B) *CLN2* and (C) *PCL1* expression levels show the same trend as in (A). (D) *SVS1* expression levels are periodic only in the WT strain while other strains with chimeric TFs show low periodic expression similar to the *swi4Δ* strain.
(TIF)

S1 Table *S*. *cerevisiae* genes that show downregulation in their expression in *swi4*Δ relative to WT, hence were considered SBF-activated genes. Genes in bold are also considered SBF targets in Ferrezuelo 2010 [12]. NA = common name not available.
(TIF)

S2 Table List of strains used in this study. (DOCX)

S1 Movie Movie showing unsynchronized division of yeast using confocal live cell imaging of *mbp1Δ* strain. Cells were immobilized to Concanavaline A (ConA) coated slides images were taken every 2 minutes for a duration of 5 hours.
(AVI)

S2 Movie Movie showing unsynchronized division of yeast using confocal live cell imaging of *mbp1Δ*, *Yl*ResBD-Swi4AD strain. Cells were immobilized to Concanavaline A (ConA) coated slides images were taken every 2 minutes for a duration of 5 hours.
(AVI)

S1 Dataset Alignment of SBF/MBF sequences. (FASTA)

S2 Dataset Gene counts from RNA seq analysis. (GZ)
