# Supplementary figures and images for: Gene duplication and co-evolution of G1/S transcription factor specificity in fungi are essential for optimizing cell fitness
Source: PLoS Genet. 2017 May 15;13(5):e1006778. doi: 10.1371/journal.pgen.1006778 (PMC5448814; doi:10.1371/journal.pgen.1006778)

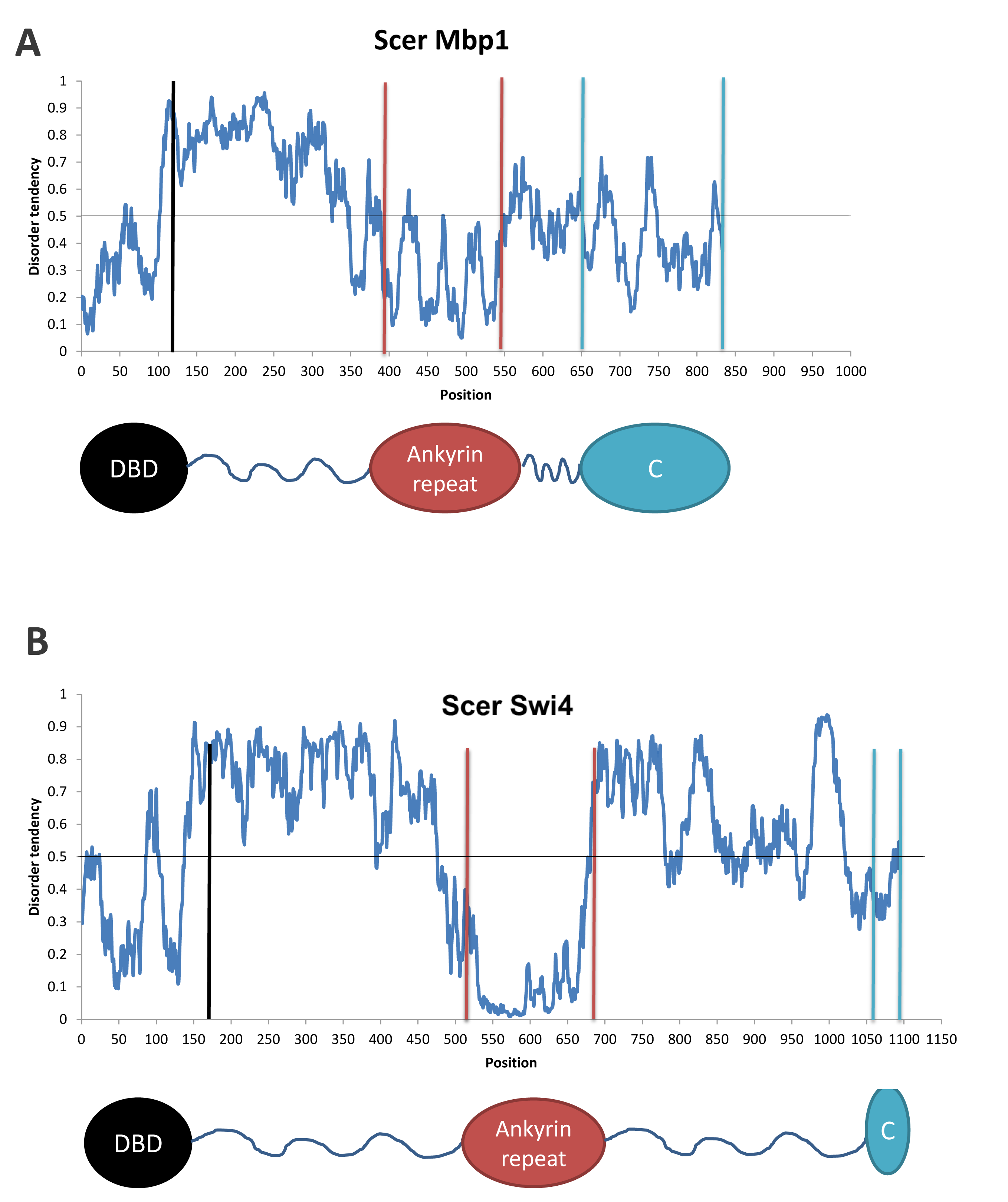

Supplement: S1 Fig — The predictor score for disorder tendency is plotted against the residue number. Residues with a higher and lower score than threshold (0.5) are considered to be in disordered and ordered regions, respectively. Large regions of the TFs are predicted to be disordered, but known functional domains including the DBD, Ankyrin repeat and the C-terminal domain are predicted to be ordered. This analysis supports a ball-and-string model where functional ordered regions are connected by flexible disordered regions. The vertical black line highlights the position of recombination for the generation of chimeric TFs. In the case of Mbp1 this is position 125 and in Swi4 this is position 165. The functional domains are annotated according to Siegmund and Nasmyth [20]. (TIF) [file pgen.1006778.s001.tif]

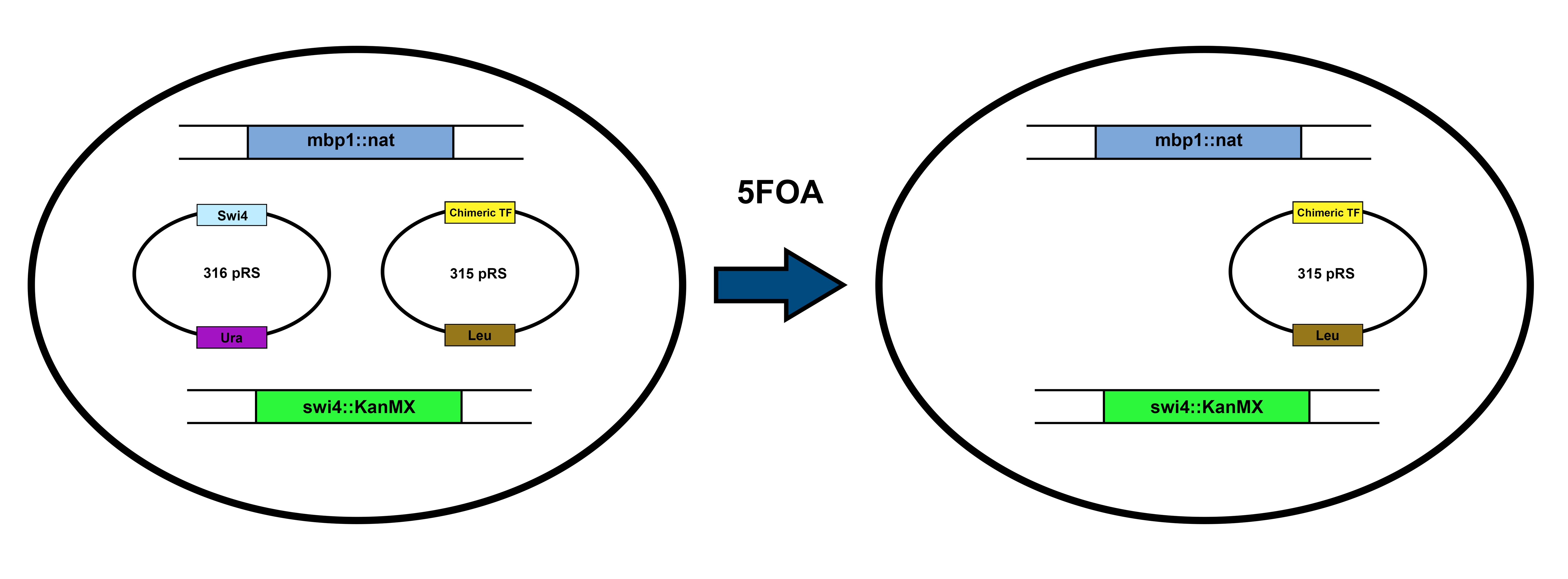

Supplement: S2 Fig — To examine the contribution of the chimeric TFs to S. cerevisiae viability we used a mbp1Δswi4Δ strain that was complemented by the WT Swi4 expressed from a URA3 centromeric plasmid and the chimeric TF expressed from a LEU2 centromeric plasmid. Upon replication of colonies on plates containing 5FOA the URA3 plasmid containing the WT Swi4 gene is lost and the plasmid encoding the chimeric TF is the sole source of Swi4 or Mbp1 expression in the cell. In cases where the chimeric TF can complement the deletions, the yeast will be viable and will grow on 5FOA plates (see Fig 2). (TIF) [file pgen.1006778.s002.tif]

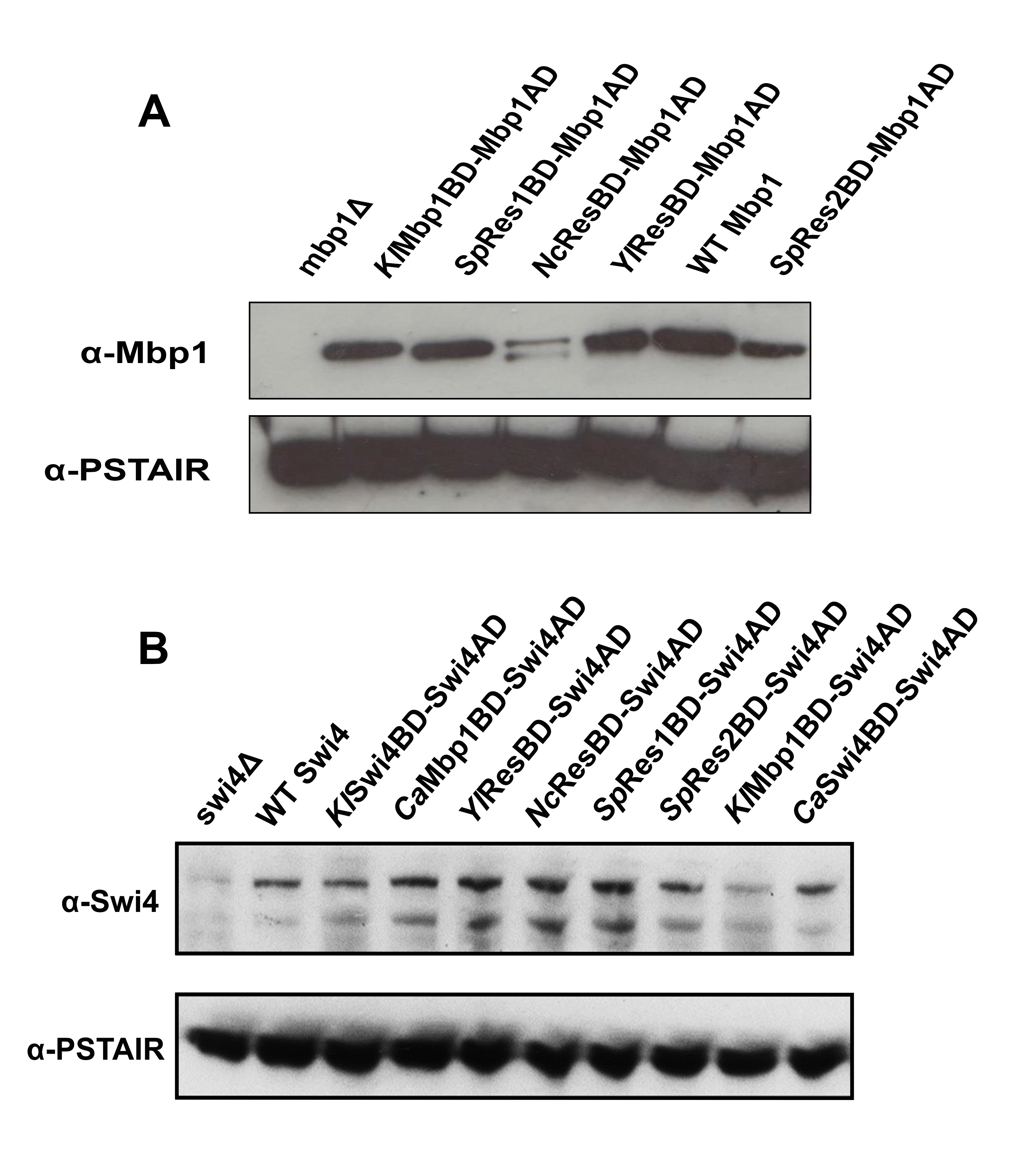

Supplement: S3 Fig — (A) Expression levels of chimeric TFs with Mbp1AD in S. cerevisiae, as assessed using α-Mbp1 antibodies. (B) Expression levels of chimeric TFs with Swi4AD in S. cerevisiae assessed using α-Swi4. The expression of PSTAIR (Cdc28p) was monitored as a loading control using α-PSTAIR antibodies. (TIF) [file pgen.1006778.s003.tif]

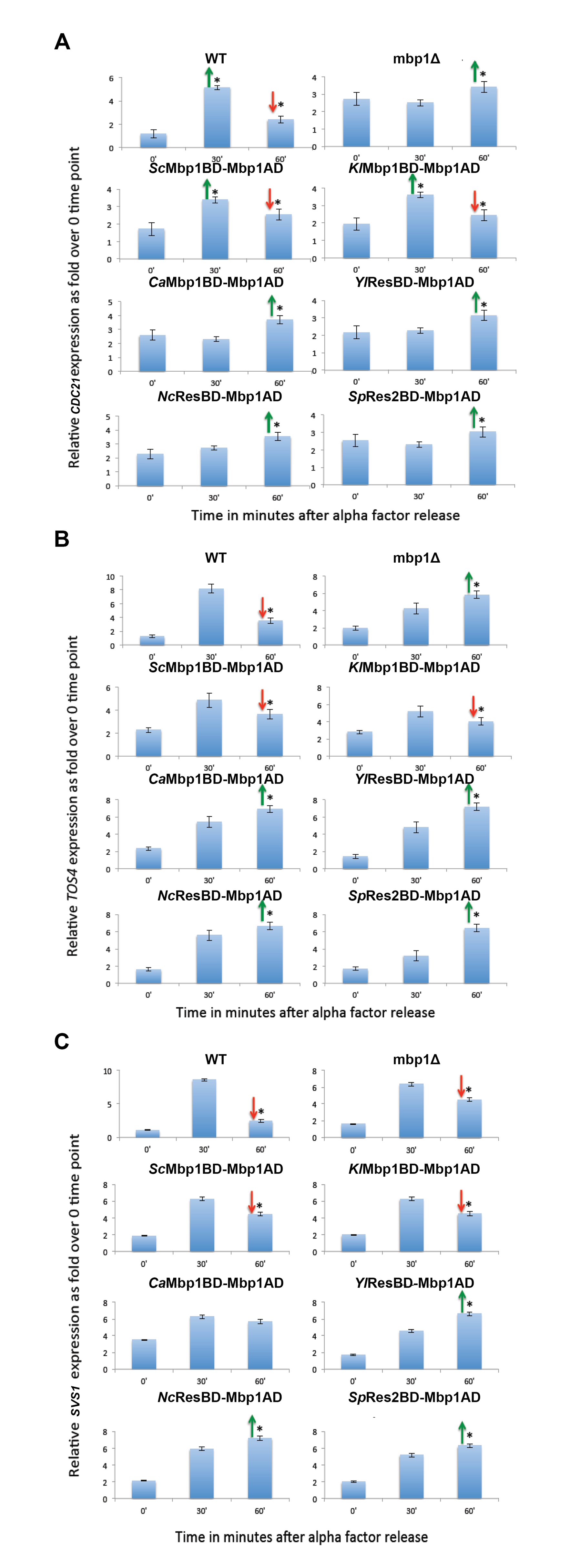

Supplement: S4 Fig — (A) Analysis of MBF target gene-CDC21 and (B) MBF and SBF target gene- TOS4 shows that only clade 1 chimeric TFs exhibit periodic gene expression. (C) SBF target gene-SVS1 analysis shows non periodic expression in strains expressing chimeric Mbp1 containing DBDs from representative strains of clades 2–5. (TIF) [file pgen.1006778.s004.tif]

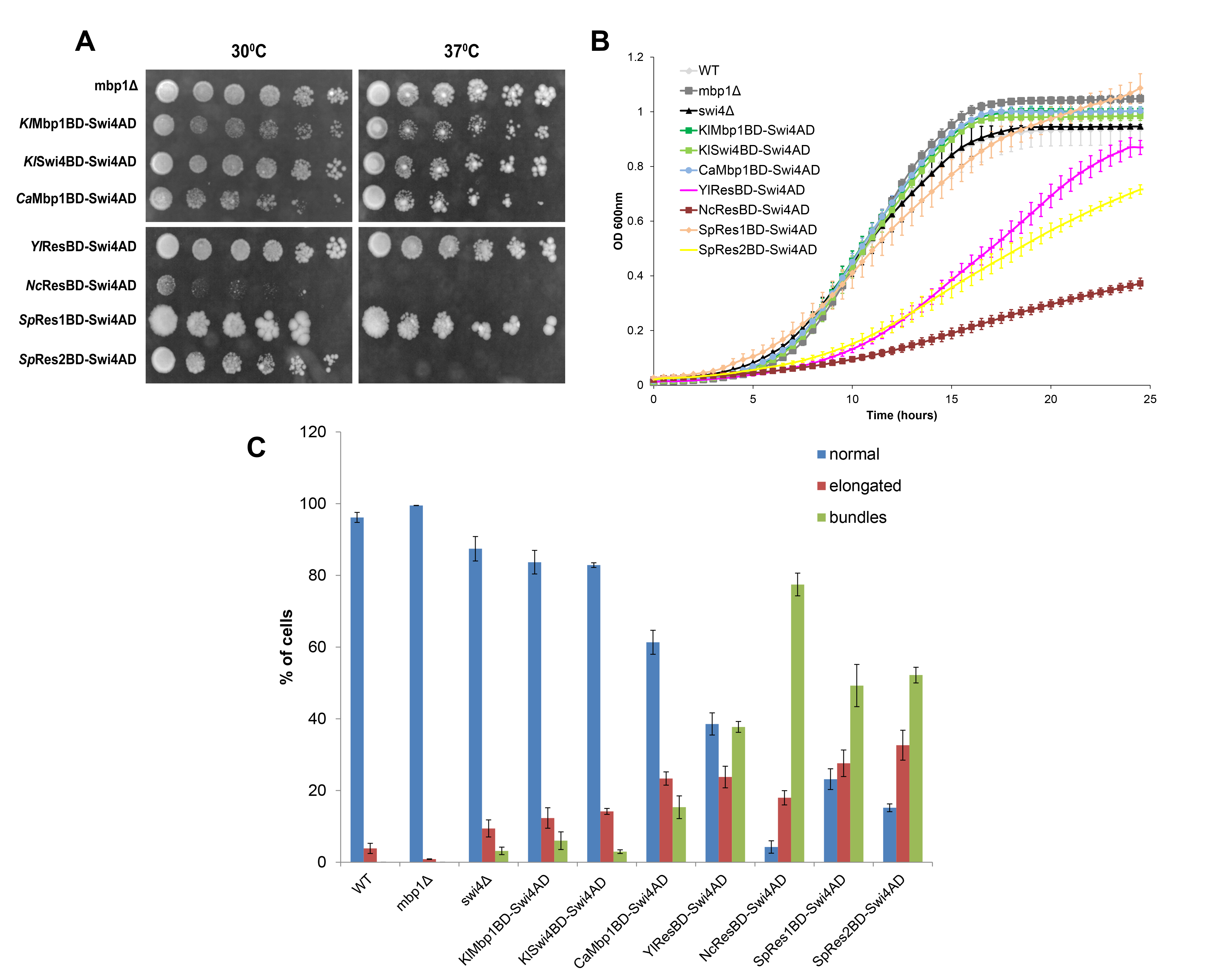

Supplement: S5 Fig — (A) Temperature sensitivity analysis of chimeric TFs with Swi4AD integrated into the S. cerevisiae genome of mbp1Δ strains. KlMbp1BD-Swi4AD, KlSwi4BD-Swi4AD, CaMbp1BD-Swi4AD and SpRes1BD-Swi4AD strains are not sensitive to restrictive temperatures (37°C), while NcResBD-Swi4AD and SpRes2BD-Swi4AD are viable only at optimal conditions (30°C). (B) Growth analysis of chimeric TFs integrated into the genome in mbp1Δ background. The strains contain KlMbp1BD-Swi4AD, KlSwi4BD-Swi4AD and CaMbp1BD-Swi4AD (Green, light green and light blue lines, respectively) display similar to the WT and mbp1Δ strain growth rate (Grey line and the dark grey line, respectively). Strains expressing the chimeric YlResBD-Swi4AD (pink line), NcResBD-Swi4AD (brown line), SpRes1BD-Swi4AD (light brown line) and SpRes2BD-Swi4AD (yellow line) exhibit decreased growth rate. (C) Quantification of the cell morphology of all strains expressing chimeric TFs as analyzed by light microscopy. (TIF) [file pgen.1006778.s005.tif]

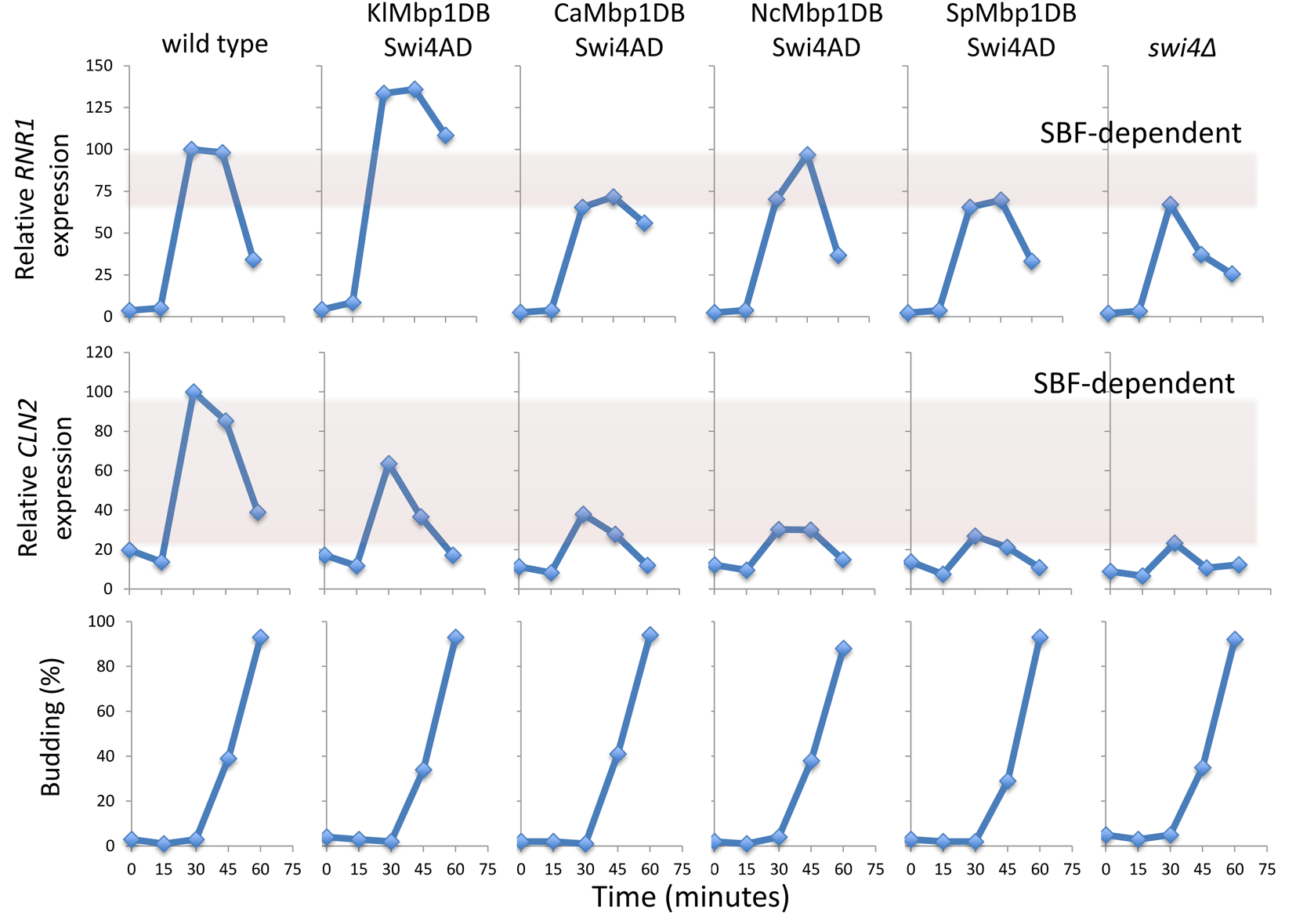

Supplement: S6 Fig — Cultures of indicated strains were synchronized by alpha factor arrest and released at time 0. Top, relative mRNA levels of RNR1 and middle, relative mRNA levels of CLN2 were analyzed by q-PCR during the cell cycle. Light red bar indicates 'SBF-dependent' transcription levels (difference between wild type and swi4Δ). Expression levels are plotted as percentage of highest value detected in wild type experiment (100%). Bottom, budding index (% budded cells) is provided as an indicator of cell cycle progression after release from alpha factor arrest. (TIF) [file pgen.1006778.s006.tif]

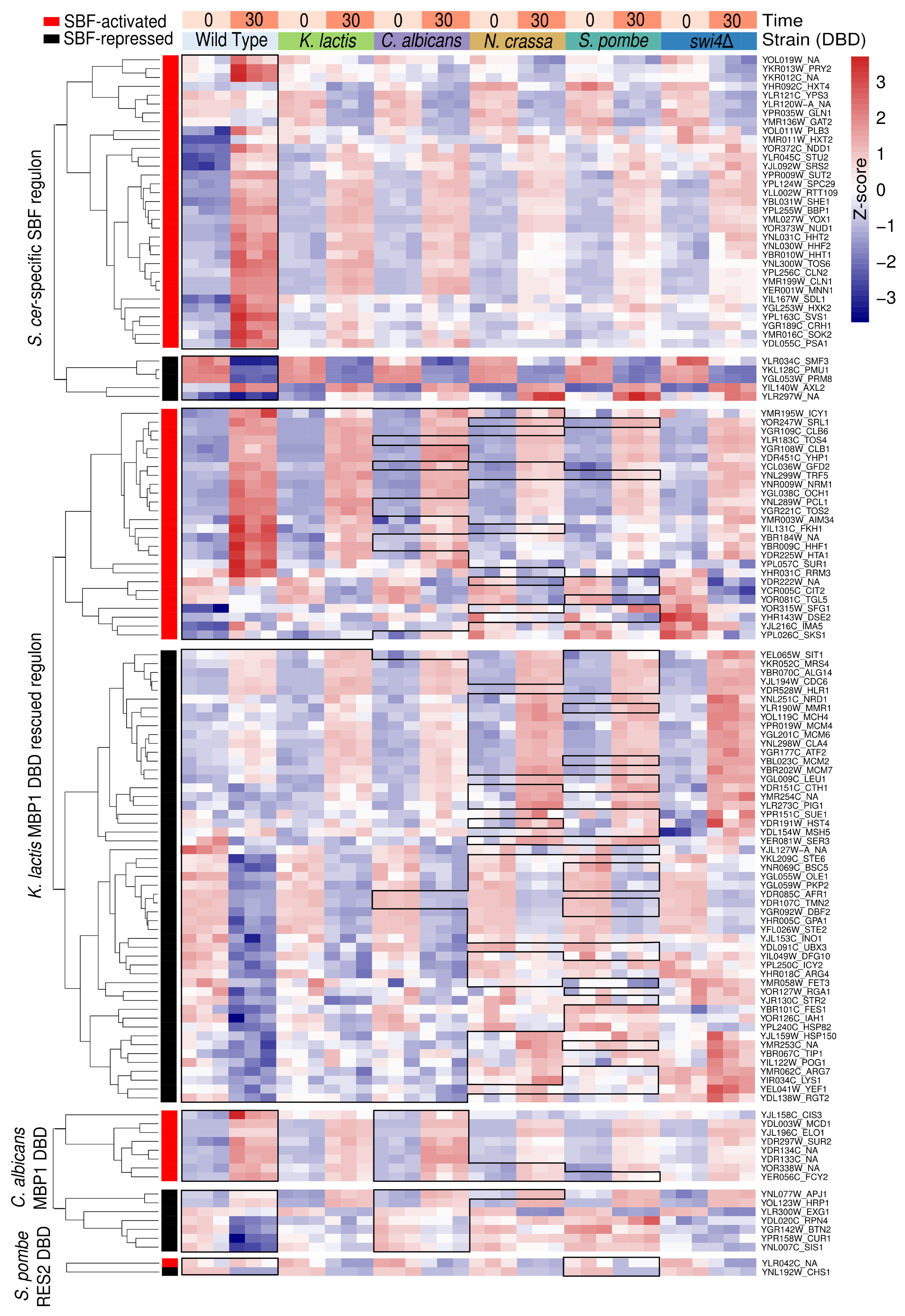

Supplement: S7 Fig — Gene expression profile of SBF-regulated genes at two time points (0 and 30 mins) after release from alpha-factor block. We plot the regularized-logarithm transformed data of each gene (row) for each strain (wild-type or DBD chimeras, columns) for visualization. All RNA-seq experiments were done in triplicate. The RNA-seq expression of each gene (heatmap) is plotted as a Z-score relative to the mean and standard deviation of all values on the same row. Statistical analysis with DESeq2 was performed on the raw data. SBF-activated genes (68 genes) and SBF-repressed genes (64 genes) are shown in red and black, respectively. Genes that had no statistical difference between RNA-seq expression at 0 and 30 mins between wild-type and chimeras were grouped together within the same contiguous box. Genes (rows) outside the box indicate that expression was significantly different from wild-type and not rescued by DBD chimeras of the corresponding species (column). Genes were organized top-down starting with the genes from SBF-regulated genes that were only rescued by ScSwi4BD (Scer-specific SBF regulon). We then list genes from SBF-regulated genes that were also rescued by KlMbp1 (Klac Mbp1BD-rescued regulon). Genes rescued by KlMbp1BD were usually rescued by CaMbp1BD with occasional rescue by NcResBD and SpRes2BD. We then show those genes not rescued by KlMbp1BD, but which were rescued by CaMbp1BD with occasional rescue by NcResBD and SpRes2BD. Last, we show those genes not rescued by KlMbp1BD, CaMbp1BD, NcResBD, but which were rescued SpRes2BD. Each module was organized using hierarchical clustering of RNA-seq profiles. (TIF) [file pgen.1006778.s007.tif]

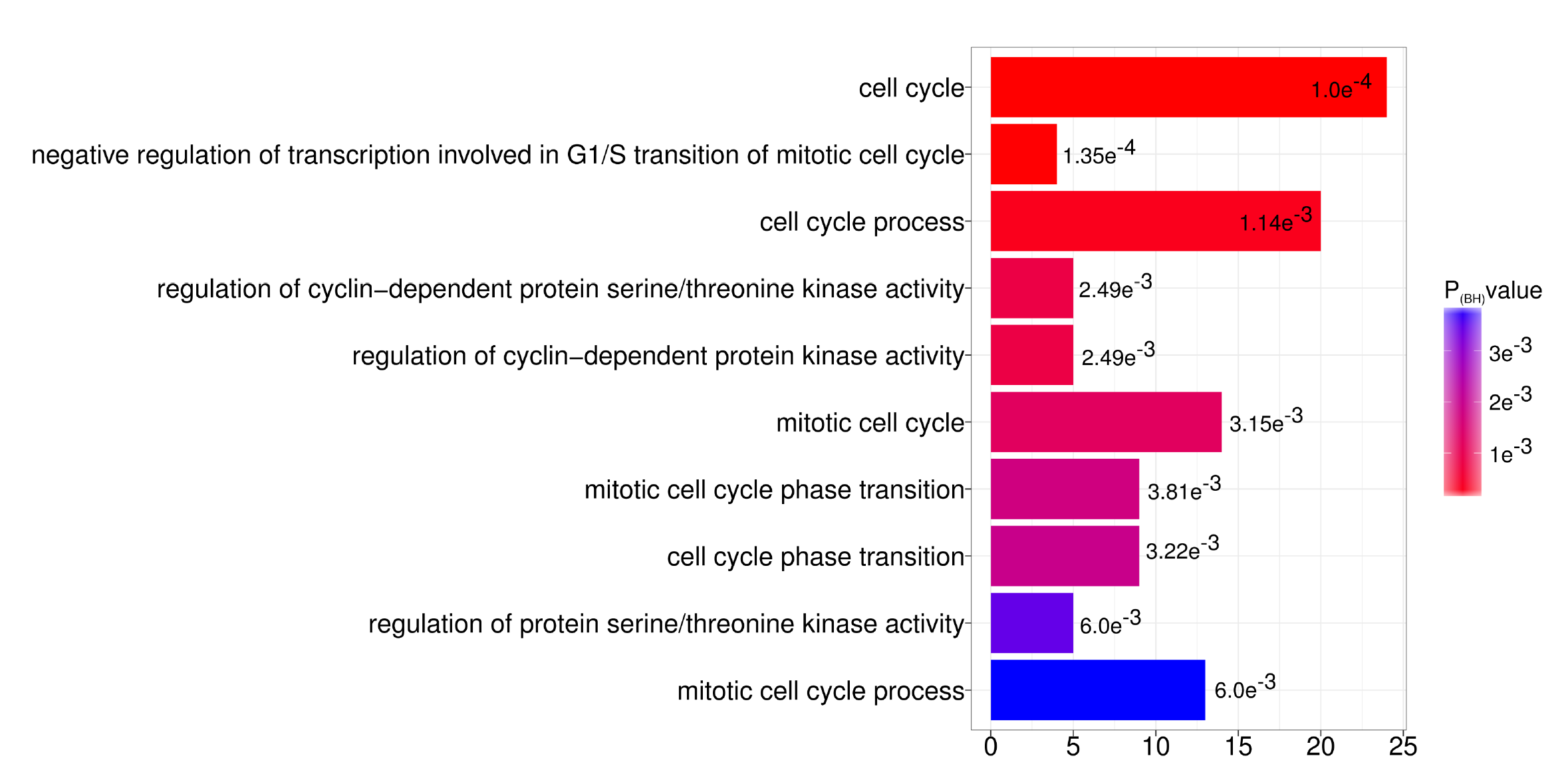

Supplement: S8 Fig — GO enrichment analysis for the SBF-activated regulon (68 genes) in “biological process” ontology terms were determined using pBH-value threshold of 0.01 and q-value threshold of 0.05. Our null model was all measured genes in our RNA-seq experiment. Length of the bar represents genes with the corresponding ontology term (e.g. 24 genes have “cell cycle” GO term), color of the bar represents enrichment pBH-value (also shown in bar). The same gene can belong to multiple GO categories. There was a total of 14 categories enriched in this dataset, but we only show the top 10. (TIF) [file pgen.1006778.s008.tif]

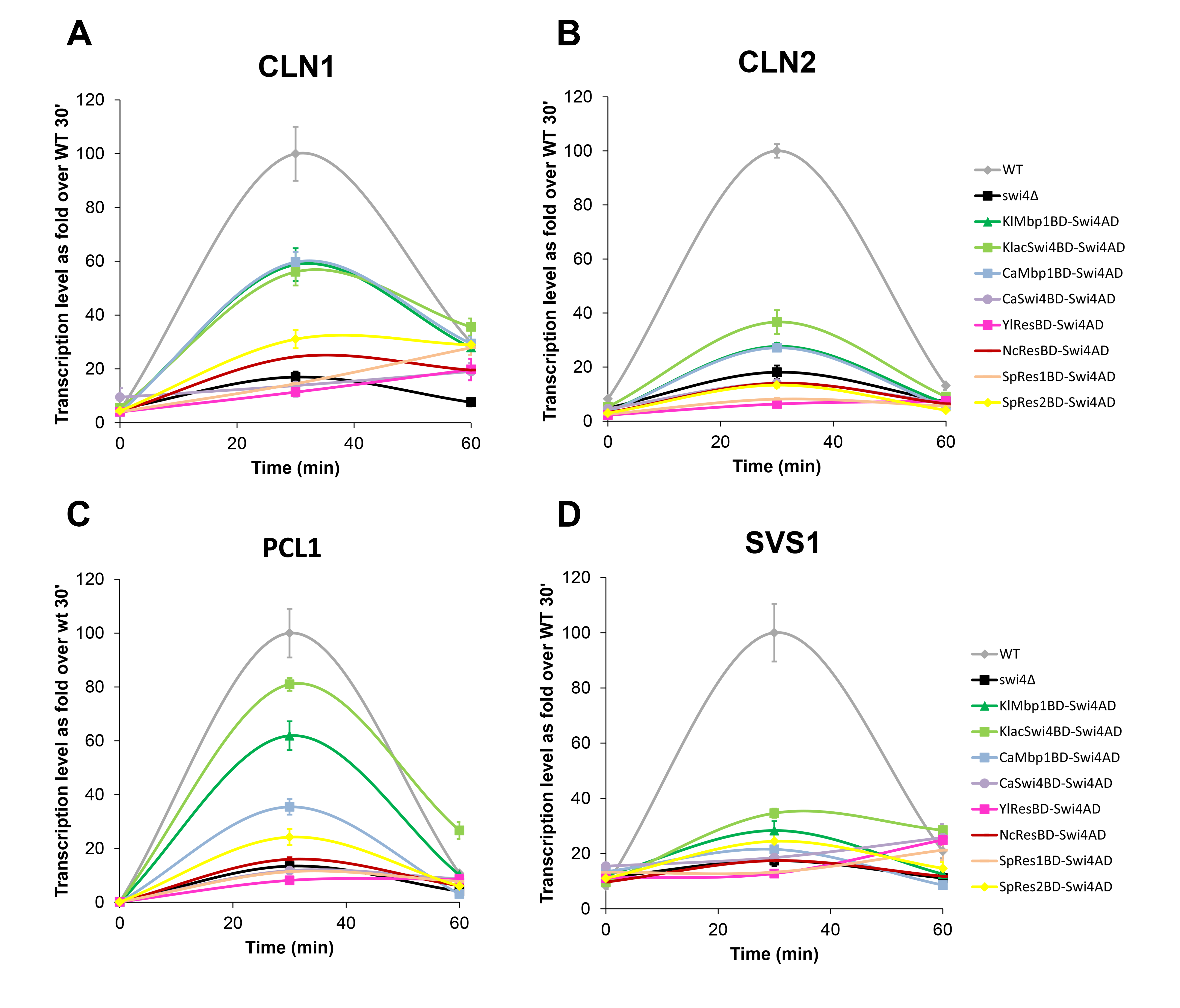

Supplement: S9 Fig — Relative expression of Swi4 target genes CLN1, CLN2, PCL1 and SVS1 was determined by qPCR analysis. (A) CLN1 expression levels in the strains containing chimeric TFs show reduced expression in more distantly related species. In strains containing KlSwi4BD, KlMbp1BD and CaMbp1BD, the gene expression of CLN1 is periodic, although peak levels are lower than in WT. Other strains with chimeric TFs show no periodic expression which is similar to the swi4Δ strain. (B) CLN2 and (C) PCL1 expression levels show the same trend as in (A). (D) SVS1 expression levels are periodic only in the WT strain while other strains with chimeric TFs show low periodic expression similar to the swi4Δ strain. (TIF) [file pgen.1006778.s009.tif]

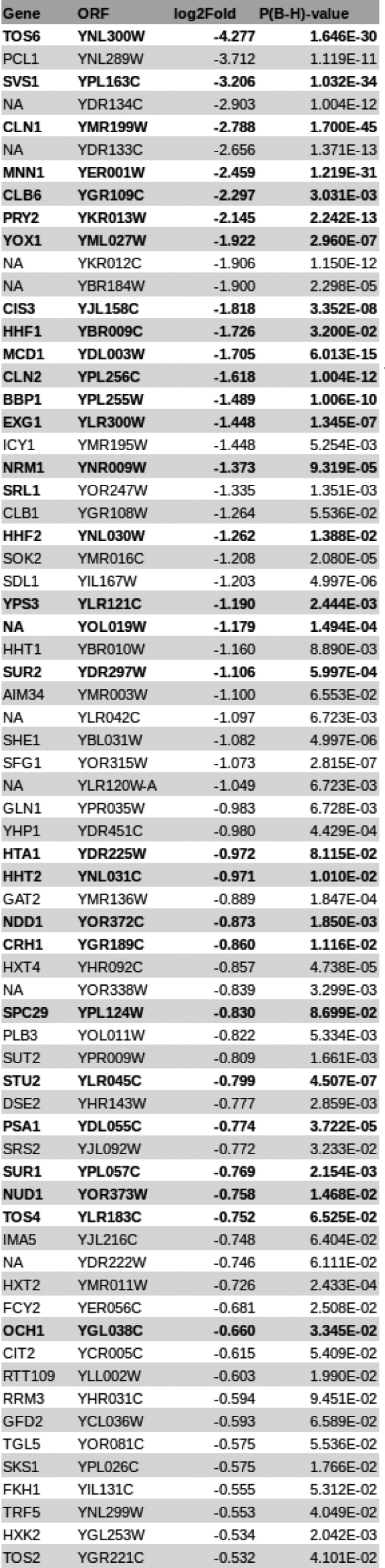

Supplement: S1 Table — Genes in bold are also considered SBF targets in Ferrezuelo 2010 [12]. NA = common name not available. (TIF) [file pgen.1006778.s010.tif]
