# Supplementary material for: Gene duplication and co-evolution of G1/S transcription factor specificity in fungi are essential for optimizing cell fitness
Source: PLoS Genet. 2017 May 15;13(5):e1006778. doi: 10.1371/journal.pgen.1006778 (PMC5448814; doi:10.1371/journal.pgen.1006778)
Supplement: S2 Table — (DOCX) [file pgen.1006778.s011.docx]

**Table S2: List of strains used in this study**

|  | Strain | Genotype | | Generated |
| --- | --- | --- | --- | --- |
| 1 | WT | 15Daub- MATa *(ade1, leu2-3, 112 his2 trp1-1 ura3* *nsΔ bar1Δ*) | | Hadwiger et al., 1989 |
| 2 | swi4Δ* | *swi4:: KanMX* | | De Bruin et al., 2006 |
| 3 | mbp1Δ* | *mbp1:: clonat* | | This study |
| 4 | *Kl*Mbp1BD-Swi4AD* | SWI4*:: KlacMbp1BD-Swi4AD::*URA3 | | This study |
| 5 | *Kl*Swi4BD-Swi4AD* | SWI4*:: KlacSwi4BD-Swi4AD::*URA3 | | This study |
| 6 | *Ca*Mbp1BD-Swi4AD* | SWI4*:: CalbMbp1BD-Swi4AD::*URA3 | | This study |
| 7 | *Ca*Swi4BD-Swi4AD* | SWI4*:: CalbSwi4BD-Swi4AD::*URA3 | | This study |
| 8 | *Yl*ResBD-Swi4AD* | SWI4*:: YlipResBD-Swi4AD::*URA3 | | This study |
| 9 | *Nc*ResBD-Swi4AD* | SWI4*:: NcraResBD-Swi4AD::*URA3 | | This study |
| 10 | *Sp*Res1BD-Swi4AD* | SWI4*:: SpomRes1BD-Swi4AD::*URA3 | | This study |
| 11 | *Sp*Res2BD-Swi4AD* | SWI4*:: SpomRes2BD-Swi4AD::*URA3 | | This study |
| 12 | *Kl*Mbp1BD-Swi4AD mbp1Δ* | SWI4*:: KlacMbp1BD-Swi4AD::*URA3 | *mbp1:: Clonat* | This study |
| 13 | *Kl*Swi4BD-Swi4AD mbp1Δ* | SWI4*:: KlacSwi4BD-Swi4AD::*URA3 | *mbp1:: Clonat* | This study |
| 14 | *Ca*Mbp1BD-Swi4AD mbp1Δ* | SWI4*:: CalbMbp1BD-Swi4AD::*URA3 | *mbp1:: Clonat* | This study |
| 15 | *Yl*ResBD-Swi4AD mbp1Δ* | SWI4*:: YlipResBD-Swi4AD::*LEU2 | *mbp1:: Clonat* | This study |
| 16 | *Nc*ResBD-Swi4AD mbp1Δ* | SWI4*:: NcraResBD-Swi4AD::*URA3 | *mbp1:: Clonat* | This study |
| 17 | *Sp*Res1BD-Swi4AD mbp1Δ* | SWI4*:: SpomRes1BD-Swi4AD::*LEU2 | *mbp1:: Clonat* | This study |
| 18 | *Sp*Res2BD-Swi4AD mbp1Δ* | SWI4*:: SpomRes2BD-Swi4AD::*URA3 | *mbp1:: Clonat* | This study |
| 19 | *PRY2*promAA | P*_PRY2_*::P*_PRY2_* with AA mutation in the SCB motif | | This study |
| 20 | *PRY2*promMCB | P*_NRM1_*::P*_PRY2_* with MCB mutation in the SCB motif | | This study |
| 21 | swi4Δ *PRY2*promAA | P*_PRY2_*::P*_PRY2_* with AA mutation in the SCB motif | *swi4:: KanMX* | This study |
| 22 | swi4Δ *PRY2*promMCB | P*_PRY2_*::P*_PRY2_*with MCB mutation in the SCB motif | *swi4:: KanMX* | This study |

***- strains created in 15D background**
